# The fidelity of DNA replication, particularly on GC-rich templates, is reduced by defects of the Fe–S cluster in DNA polymerase δ

**DOI:** 10.1093/nar/gkab371

**Published:** 2021-05-21

**Authors:** Denis A Kiktev, Margaret Dominska, Tony Zhang, Joseph Dahl, Elena I Stepchenkova, Piotr Mieczkowski, Peter M Burgers, Scott Lujan, Adam Burkholder, Thomas A Kunkel, Thomas D Petes

**Affiliations:** Department of Molecular Genetics and Microbiology, Duke University School of Medicine, Durham, NC 27710, USA; Department of Molecular Genetics and Microbiology, Duke University School of Medicine, Durham, NC 27710, USA; Department of Molecular Genetics and Microbiology, Duke University School of Medicine, Durham, NC 27710, USA; Genome Integrity and Structural Biology Laboratory, National Institute of Environmental Health Sciences, National Institutes of Health, Research Triangle Park, NC 27709, USA; Department of Genetics and Biotechnology, Saint-Petersburg State University, St. Petersburg, Russia; Vavilov Institute of General Genetics, Saint-Petersburg Branch, Russian Academy of Sciences, St. Petersburg, Russia; Department of Genetics, University of North Carolina at Chapel Hill, Chapel Hill, NC 27599-7264, USA; Department of Biochemistry and Molecular Biophysics, Washington University School of Medicine, St. Louis, MO 63110, USA; Genome Integrity and Structural Biology Laboratory, National Institute of Environmental Health Sciences, National Institutes of Health, Research Triangle Park, NC 27709, USA; Office of Environmental Science Cyberinfrastructure, National Institute of Environmental Health Sciences, National Institutes of Health, Research Triangle Park, NC 27709, USA; Genome Integrity and Structural Biology Laboratory, National Institute of Environmental Health Sciences, National Institutes of Health, Research Triangle Park, NC 27709, USA; Department of Molecular Genetics and Microbiology, Duke University School of Medicine, Durham, NC 27710, USA

## Abstract

Iron-sulfur clusters (4Fe–4S) exist in many enzymes concerned with DNA replication and repair. The contribution of these clusters to enzymatic activity is not fully understood. We identified the *MET18* (*MMS19*) gene of *Saccharomyces cerevisia*e as a strong mutator on GC-rich genes. Met18p is required for the efficient insertion of iron-sulfur clusters into various proteins. *met18* mutants have an elevated rate of deletions between short flanking repeats, consistent with increased DNA polymerase slippage. This phenotype is very similar to that observed in mutants of *POL3* (encoding the catalytic subunit of Pol δ) that weaken binding of the iron-sulfur cluster. Comparable mutants of *POL2* (Pol ϵ) do not elevate deletions. Further support for the conclusion that *met18* strains result in impaired DNA synthesis by Pol δ are the observations that Pol δ isolated from *met18* strains has less bound iron and is less processive *in vitro* than the wild-type holoenzyme.

## INTRODUCTION

Mutations must occur frequently enough to generate variants suited for evolution, but infrequently enough that essential genes are not often inactivated. In mutation-accumulation studies in *Saccharomyces cerevisiae* (1–7), the rate of single-base mutations is about 3 × 10^−10^/bp/division, and the rate of small insertion/deletions (in/dels) is at least 10-fold lower. The relatively low *in vivo* mutation rate for single-base mutations in eukaryotes is a consequence of three processes that operate sequentially: accurate base selectivity of the replicative polymerases (Pols) α, δ and ϵ; a proofreading exonuclease associated with Pols δ and ϵ, and the error-correcting mismatch repair (MMR) system (8). In addition, the catalytic subunits of DNA Pol δ and ϵ associate with other proteins (such as PCNA) that increase their processivity. These associations reduce the frequency of insertion/deletions (in/dels), mutations that likely reflect DNA polymerase slippage (9).

In yeast, as in other eukaryotes, the replicative Pols have specialized functions (8). Pol α and its co-factors synthesize short RNA–DNA fragments that are used as primers for synthesis catalyzed by the more processive Pols δ and ϵ. Although some issues remain controversial, in unstressed wild-type cells, synthesis on the leading and lagging strands is primarily performed by Pols ϵ and δ, respectively; however, in certain mutant backgrounds and in certain genomic regions in wild-type cells, Pol δ replicates both leading and lagging strands (10,11).

Pol δ in *Saccharomyces cerevisiae* is composed of three subunits: Pol3p which has the catalytic activities of the enzyme (12), and the accessory subunits Pol31 and Pol32 (13,14). Pol3p and Pol31p are encoded by the essential yeast genes *POL3* and *POL31*, respectively. *POL32* is not essential. Previous studies showed that point mutations or low levels of Pol3 substantially increase mutation rates in yeast, particularly the rates of multi-base deletions flanked by short direct repeats (15,16). Amino acid substitutions in *POL3* that reduce base selectivity or eliminate the proofreading exonuclease, as expected, have a strong mutator phenotype (3,17).

Pol α in *S. cerevisiae* is composed of four essential proteins: Pol1 (catalytic subunit), Pol12 (B subunit of the complex), and two proteins with primase activity, Pri1 and Pri2 (8). Pol ϵ has four subunits encoded by the yeast genes *POL2*, *DPB2*, *DPB3* and *DPB4* (8). Although strains with a deletion of *POL2* are not viable, strains that have a deletion of Pol2p that removes the catalytic region of the protein are viable (18) as a consequence of the ability of Pol δ to substitute for ϵ in replicating the leading strand (11).

Pol3p was shown to have an iron-sulfur (Fe–S) cluster of the 4Fe–4S type coordinated within the protein by four cysteine residues (19,20) located close to its C terminus. While the exact role of this metal cofactor in DNA polymerases is not completely understood, *in vitro* studies showed that the redox state of the Fe–S cluster can control the rate of DNA synthesis catalyzed by Pol δ (21). Mutations of the Fe–S coordinating cysteine residues strongly reduce iron binding to Pol3 (19) and some of these mutants are inviable or thermo-sensitive *in vivo* (22). These observations strongly suggest an important role of the Fe–S cluster for the proper functioning of the Pol δ. Since cysteine mutants of Pol3 prevent the binding of the accessory subunits Pol31 and Pol32 (19), one may attribute some of the effects of these mutants to the absence of these accessory proteins.

A well-studied cysteine mutation *pol3–13* (22) is located within the Fe–S cluster binding domain of Pol3p. The mutation results in thermosensitivity, and is synthetically lethal with some other mutations, including *met18/mms19* (23). *MET18* (*MMS19*) encodes a component of the cytosolic iron-sulfur cluster assembly (CIA) machinery (24) and is responsible for the introduction of the Fe–S cluster into target proteins.

As described below, we identified a mutation that had a substantial mutator phenotype with particularly strong effects on GC-rich genes. We previously demonstrated (25) that a *URA3* gene constructed to be GC-rich had a seven-fold elevated rate of mutation compared to a ‘normal’ *URA3* gene. We find that the *met18* mutation elevates the mutation rate of a GC-rich gene about 100-fold relative to a wild-type strain, and elevates mutation rates about 7-fold throughout the genome. Almost all of the mutations induced in GC-rich genes are deletions between short direct repeats, suggesting that they arise through DNA polymerase slippage. Further, we show that the mutator phenotype of the *met18* strain is likely a consequence of loss of the Fe–S cluster from Pol δ. We also demonstrate that Pol δ isolated from *met18* strains binds less iron than that isolated from wild-type strains, and is less processive *in vitro*.

## MATERIALS AND METHODS

### Strain constructions and growth conditions

Most of the yeast strains used in these experiments were derivatives of W1588–4C (26), a *RAD5* derivative of W303-1A (27). Derivatives of strains JED213-30 (15) and BJ2168 (ATCC^®^ 208277™) were used for protein analysis. Strain MD702 is a derivative of YJM789 (28). The genotypes of each strain and the details of their constructions are in Supp. Expt. Procedures and in Supplementary Table S1. Plasmids and primers used for these constructions are in Supplementary Table S2. Standard media were used, including rich (YPD), sporulation media, synthetic complete (SC), and synthetic complete lacking one or more components (29).

Gene deletions were made by single-step transplacement with PCR-generated fragments encoding either the *natMX4* or *hphMX4* cassettes (30), and were selected on plates containing YPD with nourseothricin (100 mg/l) or hygromycin B (300 mg/l), respectively. Point mutations were introduced into chromosomal copies of *POL1*, *POL2* and *POL3* by two-step plasmid integration and excision (31).

The *CAN1-GC* sequence was synthesized by GenScript. Sanger sequencing was performed by Eton Bioscience. Oligonucleotides used in this study are listed in Supplementary Table S2 and were synthesized by Eton Bioscience or Integrated DNA Technologies. Site-directed mutagenesis was performed by using Q5 Site-Directed Mutagenesis Kit (New England BioLabs) or the QuikChange Site-Directed Mutagenesis Kit (Agilent) according to the manufacturer's instructions.

Canavanine-resistant colonies were selected on SC-Arg media with 120 mg/l canavanine added. Ura^−^ colonies were selected on SC media with 0.1% of 5-fluoro-orotate (5-FOA). Strains bearing the following mutations were temperature-sensitive, and were grown at 23°C: *pol3–13*, *pol3-C1069S*, *pol2-C763S* and *pol2-C665S,C677S*. All other strains were grown at 30°C unless otherwise indicated.

### Determination of mutation rates and mutation analysis

To estimate *CAN1* and *URA3* mutation rates, we performed fluctuation analysis. Multiple (about 20) single colonies derived from each strain were isolated from YPD plates and patches were made from each colony on YPD. After two-three days of growth, the resulting patches were suspended in sterile water. The appropriate dilutions were then grown on selective plates containing canavanine or 5-FOA to determine the rate of *CAN1* or *URA3* mutations, and on non-selective plates to determine the total number of cells in the patch. Plates were scored after three days of incubation at 30°C for most strains or after five days of incubation at 23°C for temperature-sensitive strains. Mutation rates were calculated using the method of the median (32). VassarStats tools (http://www.vassarstats.net/) were used to produce confidence intervals for proportions and for the chi-square test. Confidence intervals for individual mutation events were calculated as described previously (25).

Mutated *URA3* genes were isolated by PCR amplification of DNA isolated from independent 5-FOA^R^ isolates using the primers DKo3 and DKo4 (Supplementary Table S2–2), and the resulting product was sequenced using the same two primers. Mutant *CAN1* genes were amplified from independent Can^R^ isolates using the primers DKo74 and DKo79 (Supplementary Table S2–2). The resulting fragments were sequenced using these two primers plus the primers DKo76 (*CAN1-WT*) or DKo77 (*CAN1-GC*) and DKo78.

For mutation-accumulation experiments, DNA samples were prepared, sequenced (paired-end Illumina sequencing with HiSeq 4000), and analyzed as described in (33). Mutations were detected and classified using ‘muver’ (*mutations verificatae*) software as described in (34). Data from the wild-type strain (TAK948) are archived on the GEO database (https://www.ncbi.nlm.nih.gov/geo/query/acc.cgi?acc=GSE101698) listed as GSM2712335. Sequencing data for the wild-type strain delta|(–2)|-7B-YUNI300 (33) is archived at: (https://www.ncbi.nlm.nih.gov/geo/download/?acc=GSE56939) (LO3_ref_v2). The data from the *met18* strain DKy361 are located on the Sequence Read Archive database (BioProject ID: PRJNA703018).

### Mapping the *met18-410* mutation using DNA microarrays

As described in the Results, we crossed DKy240 (the haploid strain containing the mutator mutation) to an isogenic wild-type strain of the opposite mating type (DKy97) to form the diploid DKy309. When the heterozygous diploid was sporulated, the phenotype of the elevated mutation rate of *URA3-GC* segregated 2:2 in 10 of 10 tetrads. This result indicates that the mutator phenotype is a consequence of a single mutation in DKy240. To identify the mutation, we crossed DKy240 to MD702, a haploid strain of the opposite mating type. The MD702 strain was derived from the strain YJM789 that contains about 55 000 single-nucleotide polymorphisms (SNPs) relative to the W303-1A background of DKy240 (35). Following tetrad dissection of the resulting diploid (DKy359), we screened the haploids derived from the spores for those that produced 5-FOA^R^ colonies at a high rate.

We then used SNP-specific microarrays to determine which genomic sequences within each haploid strain were from the W303–1A-derived homolog or the YJM789-derived homolog (35,36). Of the ten meiotic segregants that had the mutator phenotype (examined in five pools of two segregants), all ten shared the same 21-kb segment of chromosome IX of the W303–1A-derived genotype; no other genomic segment was shared among the ten mutator strains. An example of the analysis is shown in Supplementary Figure S2. This region contains five genes: *ASG1*, *TAO3*, *MET18*, *RRT14* and *STH1*. A mutation in *MET18* would be expected to result in a Met^−^ phenotype, and we found that all spores that had a high frequency of mutations for *URA3-GC* were Met^−^. Subsequently, by sequence analysis, we showed that the Met^−^ meiotic products had a frameshift mutation (AT to G in position 2816–2817) in *MET18*. This mutant allele (which we called ‘*met18–410C*’) was absent from the unmutagenized parental strains.

### Isolation and analysis of Pol δ

To evaluate levels of (3xHA)-Pol3, we used the following protocol. Cells were grown in liquid YPD to an OD_600_ of 0.4–0.5, harvested by centrifugation, and washed twice with water. Cells were broken by agitation with acid-washed glass beads in the lysis buffer (10% glycerol, 20mM Tris pH8, 1 mM EDTA pH 8, 150 mM NaCl, 1× cOmplete™ Mini EDTA-free Protease Inhibitor Cocktail (Roche)). Lysates were cleared by centrifugation at 10 000g for two minutes, and then mixed with 4× sample buffer (40% glycerol, 280 mM Tris pH 6.8, 4% SDS, 0.02% bromophenol blue, 2% 2-mercatoethanol). Proteins were separated on a Novex™ WedgeWell™ 4–12% Tris–glycine Mini Protein Gel (Thermo Fisher Scientific), and transferred to an Invitrolon™ PVDF (Thermo Fisher Scientific) membrane. Membranes were probed with anti-HA antibody (ab9110, Abcam) or an anti-GAPDH antibody (ab9385, Abcam).

Multi-subunit DNA polymerase δ purification was adapted from (37–40). Briefly, overexpression of proteins was performed in the strain BJ2168 or its *met18Δ* derivative (DKy501). For Pol δ holoenzyme overexpression, these strains were transformed with pBL341 and pBL335, grown in SCGL media (SC with 1 g/l glucose, 30 g/l glycerol, 20 g/l lactic acid as carbon sources) without uracil and tryptophane followed by induction with equal volume of 4% galactose YPGL media (YPD with 2 g/l glucose, 30 g/l glycerol, 20 g/l lactic acid, 40 g/l galactose as carbon sources) for 6 h. Pelleted cells were resuspended in water (cells to water ratio 2:1), frozen in liquid nitrogen and lysed by freezer mill. Lysed cells were thawed and solubilized 2:1 in 3X Hep150 buffer (90 mM Hepes, pH 7.8, 150 mM NaCl, 30% glycerol, 6 mM EDTA, 0.06% NP40, 6 mM DTT, 30 mM NaHSO_3_, 30 μM pepstatin A, 30 μM leupeptin) with 1 mM PMSF, to which ammonium sulfate and polymin-P were added in two steps to 150 mM and 45 μl/ml, respectively. The solution was spun in the ultracentrifuge, 18 000 RPM for 30 minutes at 4°C.

The supernatant was precipitated with 0.3 g/ml ammonium sulfate over 1 h at 4°C. The pellet was collected by ultracentrifuging at 18 000 RPM for 30 min at 4°C and resuspended in 1× Hep0. Solubilized pellet was batch bound overnight at 4°C to 2 ml Hep250 equilibrated glutathione Sepharose 4B beads. The slurry was washed by gravity filtration with 35 mL Hep250, 10 mL Hep250 containing 1 mM ATP and 3 mM MgOAc, 10 mL Hep150, then 10 mL Hep50. The purified DNA polymerase δ complex was washed from beads at 4°C with 9 ml Hep50 + 20 mM glutathione (pH 8.0) and collected overnight into conical tube on ice containing 20U PreScission Protease. Extra purification was performed by FPLC using a MonoS column over a 15 mL linear gradient of 50 mM NaCl and 1000 mM NaCl solutions containing 30 mM HEPES pH 7.4, 10% glycerol, 1.5 mM EDTA, 0.01% NP40, 5 mM DTT, 2 μM pepstatin A, 2 μM leupeptin, 0.1% ampholytes 3.5–9.

Analysis of the polymerizing and exonuclease activities of Pol δ were done as described in (41). In brief, a Cy3-labeled oligonucleotide used for the primer (5′-/5Cy3/AAAAATTGTACTTGGCGGATAATGCCTTTAGCGGCTTAACT) was annealed to a template oligonucleotide (5′-GGATATCTTGACTGATTTTTCCATGGAGGGCACAGTTAAGCCGCTAAAGGCATTATCCGCCAAGTACAATTTTT) by heating at 94°C then slow cooling in a heat block to 30°C. 15 μl reactions consisting of 50 nM DNA hybrid, 50 nM Enzyme (WT or Met-18 Pol δ), cellular concentrations of each of the four dNTPS (dA, 16 μM; dC, 14 μM; dG, 12 μM; dT, 30 μM) 1mM DTT, 5% glycerol, 1xTE were preincubated for 10 min at 30°C. These reactions were initiated by the addition of 2 μl of a solution containing 11 mM MgCl_2_, 4μg/ml heparin and stopped after 2 min by the addition of equal volume 95% formamide, 20 mM EDTA, 0.01% bromophenol blue. 10 μl of these stopped reactions were analyzed by denaturing 12% polyacrylamide gels, and quantified with ImageQuant.

The iron content in samples with FPLC-purified Pol3 was determined by inductively coupled plasma mass spectrometry (ICP-MS), and was performed by RTI International at Research Triangle Park, NC.

### Yeast two-hybrid analysis

The interaction between Pol3 and Pol31 was analyzed by yeast two-hybrid assays carried out in the wild-type strain L40 (*MAT***a**, *his3*-Δ*200*, *trp1–901*, *leu2–3,112*, *ade2*, *LYS2*::(lexAop)_4_-*HIS3*, *URA3*::(lexAop)_8_-lacZ) or the isogenic strain DKy440, a *met18Δ* derivative of L40. The strains L40-pBL322/pBL364 and DKy440-pBL322/pBL364 were used to measure Pol3–Pol31 interactions with the plasmids pBL322 (LexABD-*POL3* in 2μ vector pBTM116 with *TRP1* marker) and pBL364 (*GAL4*AD-*POL31* in 2μ vector pACT2 with *LEU2* marker). In strains L40-pBL323/pBL363 and DKy440-pBL323/pBL363, the interactions were measured with the plasmids pBL323 (*GAL4*AD-*POL3* in 2μ vector pACT2 with *LEU2* marker) and pBL363 (LexBD-*POL31* in 2μ vector pBTM116 with *TRP1* marker). The plasmids used in this analysis are described in (13). Quantitative β-galactosidase assays were carried out in triplicate.

## RESULTS

### Identification of *met18* as a strong mutator biased toward mutagenesis of a GC-rich gene

In our previous study (25), we showed that a gene with a high-GC content (*URA3-GC*, 63%) had an elevated rate of both mutations and recombination relative to a gene with a more average GC content (*URA3-WT*, 43%); the GC-content of the nuclear genome of *S. cerevisiae* is about 38% (42). We found that the high mutation rate of the *URA3-GC* allele was a consequence of a strongly elevated rate of deletions between direct repeats (likely a consequence of DNA polymerase slippage by the replicative Pols) as well as an elevated rate of single-base mutations caused by increased recruitment of the error-prone Pol ζ. Based on these observations, we performed a mutant hunt to look for variants that had an elevated rate of mutations for the *URA3-GC* template but lesser effects on the rate of mutations in a gene with a lower GC content (41%, *CAN1*).

The haploid strain DKy232–8C (isogenic except for alterations introduced by transformation with W303–1A (27)) was mutagenized with ultraviolet light with a dose (20 J/m^2^) that reduced viability to about 50% (Supplementary Figure S1). DKy232–8C has two markers that were used to screen for mutations, *CAN1* (41% GC-content) and *URA3-GC* (63% GC-content). Mutations in *CAN1* and *URA3-GC* result in derivatives that are resistant to canavanine and 5-fluoro-orotate (5-FOA), respectively. Following mutagenesis, the cells were allowed to form colonies on a rich growth medium, and the resulting colonies were replica-plated onto medium containing canavanine or 5-FOA. Out of approximately 14,000 colonies tested, a single isolate (DKy240) reproducibly had 26-fold more colonies than the unmutagenized isogenic strain on plates containing 5-FOA, but only a 4-fold increase in colonies resistant to canavanine (details in Materials and Methods).

We crossed DKy240 to an isogenic wild-type strain of the opposite mating type (DKy97) to form the diploid DKy309. When the heterozygous diploid was sporulated, the phenotype of the elevated mutation rate of *URA3-GC* segregated 2:2 in 10 of 10 tetrads, indicating that the mutator phenotype was a consequence of single mutation in DKy240. To identify this mutation, we crossed DKy240 to MD702, a haploid strain of the opposite mating type that had about 55 000 single-nucleotide polymorphisms (SNPs) relative to DKy240 (35). We used SNP-specific microarrays to determine which SNPs co-segregated with the mutator phenotype in spores derived from the diploid (details in Supp. Expt. Procedures, Supplementary Figure S2). Subsequently, by sequence analysis, we showed that the meiotic products with a mutator phenotype had a frameshift mutation (AT to G in position 2816–2817) in *MET18*. This mutant allele (*met18–410C*) was absent from the unmutagenized parental strains.

We then determined the effect of *met18–410C* on *URA3-WT* (43% GC) in an isogenic strain in which *URA3-GC* (63% GC) was replaced by *URA3-WT* (DKy242–1C; Supplementary Tables S1 and S2). We found that the mutation rate of *URA3-WT* was elevated by about 10-fold instead of the 26-fold elevation observed for *URA3-GC* (Table 1). Thus, the *met18* mutator phenotype is preferentially associated with the high-GC gene.

**Table 1.** Rates of different types of *URA3* and *CAN1* mutations in strains with mutations in *met18* or various DNA polymerase genes

Genotype	Assayed gene	Temp. (°C)	Total rate of mutations (μ x 10^−9^)	Rates of single base mutations (μ x 10^−9^)	Rates of deletions (insertions) ≥ 5 bp (μ x 10^−9^)	Rates of deletions (insertions) <5bp (μ x 10^−9^)	Rates of *ura3-GC-366TC* mutations (μ x 10^−9^)	Rates of other mutations (μ x 10^−9^)
WT	*URA3-GC*	30	49.4	25.5	12.5 (3.3)	5.4 (<0.5)	<0.5	2.7
WT	*URA3-GC*	23	87.5	15.7	55.1 (4.9)	7.9 (<1.0)	<1.0	3.9
*met18–410C*	*URA3-GC*	30	1270	<29	1100 (<29)	<29 (<29)	174	<29
*met18-Δ*	*URA3-GC*	30	6580	<88	5350 (<88)	<88 (<88)	1050	176
*met18-Δ mlh1-Δ*	*URA3-GC*	30	12700	4870	3900 (<325)	1620 (<325)	2270	<325
WT	*URA3-WT*	30	7.3	5.9	<0.1 (<0.1)	0.6 (0.1)	NR	0.7
*met18–410C*	*URA3-WT*	30	75.4	18.1	48.7 (1)	2.9 (1)	NR	3.8
*met18-Δ*	*URA3-WT*	30	259	74.1	148 (12.3)	8.2 (<4.1)	NR	16.5
*met18-Δ rev3*	*URA3-WT*	30	182	38.3	133 (<1.7)	1.7 (<1.7)	NR	8.3
*WT*	*CAN1-WT*	30	188	135	6.7 (3.4)	26.9 (6.7)	NR	10.1
*met18–410C*	*CAN1-WT*	30	694	277	173 (<35)	104 (104)	NR	34.7
*met18-Δ*	*CAN1-WT*	30	1160	172	882 (<21)	21.5 (<21)	NR	86.1
*WT*	*CAN1-GC*	30	424	277	29.4 (<4.2)	84 (4.2)	NR	29.4
*met18-Δ*	*CAN1-GC*	30	6440	<105	6340 (<105)	106 (<105)	NR	<105
*pol3-C1059S*	*URA3-GC*	30	8920	121	8200 (<121)	0 (<121)	603	<121
*pol3-C1059S*	*URA3-WT*	30	261	106	131 (2.8)	5.6 (5.6)	NR	11.1
*pol3-C1059S*	*CAN1-WT*	30	1160	504	504 (21.9)	110 (<22)	NR	21.9
*pol3–13*	*URA3-GC*	23	8020	108	5530 (<108)	108 (<108)	1950	325
*pol3-C1069S*	*URA3-GC*	23	3480	256	2620 (32)	32 (32)	448	63.9
*pol3-C1059S*	*URA3-GC*	23	2390	104	1975 (<52)	<52 (<52)	312	<52
*met18-Δ*	*URA3-GC*	23	4410	74.8	3740 (<75)	<75 (<75)	524	74.8
*pol2-C677S*	*URA3-GC*	30	47.6	19.9	13.0 (4.3)	10.4 (<0.9)	<0.9	<0.9
*pol2-C677S*	*URA3-GC*	23	84.4	27.0	40.9 (7)	5.2 (0.9)	<0.9	3.5
*pol2-C2181S*	*URA3-GC*	30	33.4	12.5	14.6 (1.6)	4.7 (<0.5)	<0.5	<0.5
*pol2-C763S*	*URA3-GC*	23	456	248	65.2 (8.7)	86.9 (8.7)	8.7	30.4
*pol2-C665S/C677S*	*URA3-GC*	23	539	265	108 (19.6)	78.4 (9.8)	<9.8	58.8
*pol32-Δ*	*URA3-GC*	23	2110	49.7	1810 (<25)	<25 (25)	224	24.9
*pol32-Δ*	*URA3-GC*	30	455	49	308 (7.0)	<7 (<7)	90.9	<7

The entries in this table summarize the details presented in Supplementary Table S3. In the columns showing the rates of deletions and insertions, the insertion rates are in parentheses. As discussed in the text, the *ura3-GC-366TC* mutation represents conversion of a quasi-palindrome sequence into a perfect palindrome. ‘NR’ indicates that the rate of *ura3-GC-366TC* mutations is not relevant because the reporter gene does not contain a quasi-palindrome. For categories in which the number of events was 0, we calculated the rate as less than the value obtained if there was a single event.

### Mutator phenotype in *met18Δ* strains

**Figure 1.**
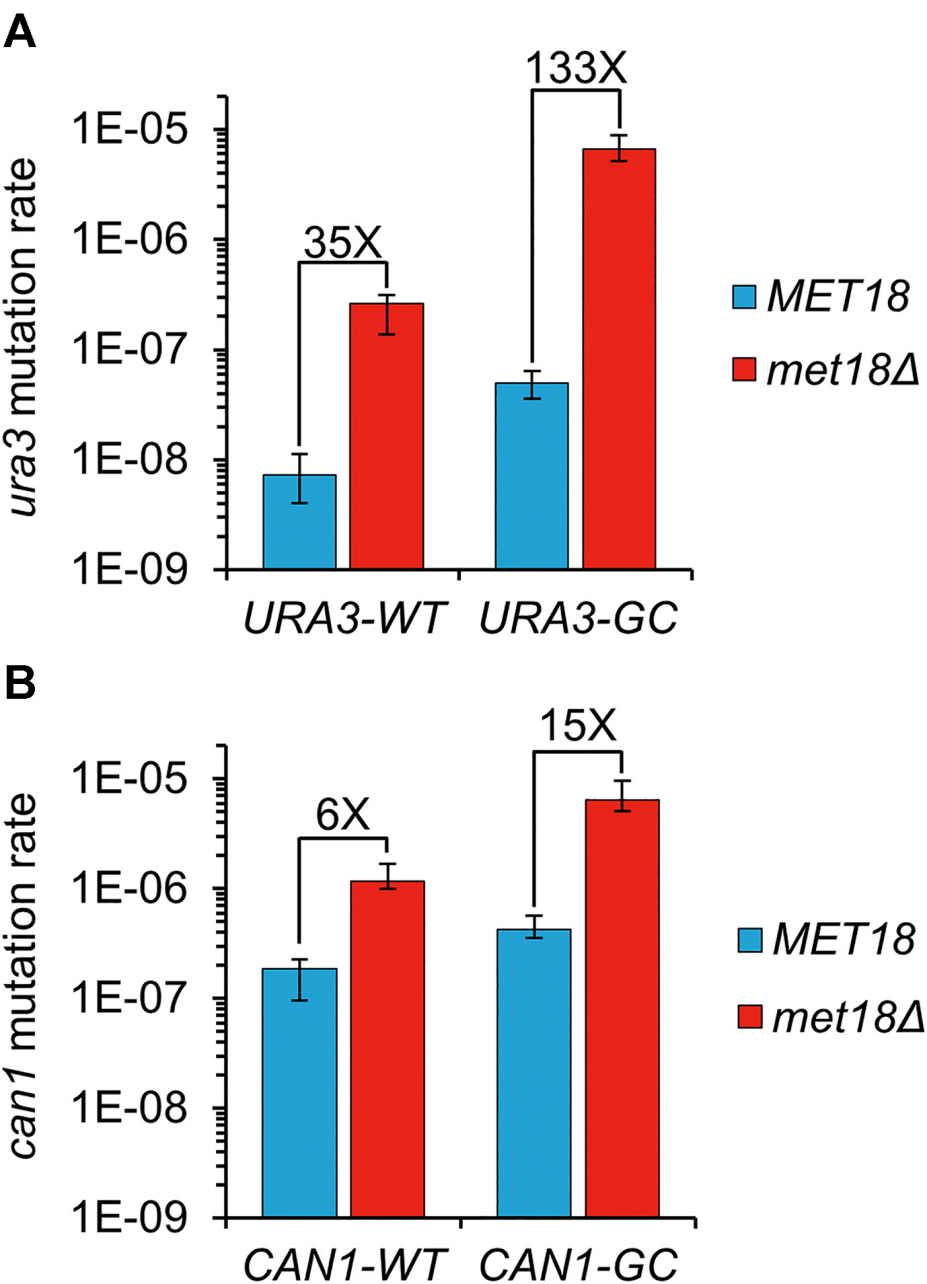
Mutator phenotype of *met18Δ* on genes with wild-type GC-content (*URA3-WT* and *CAN1-WT*), and GC-rich genes (*URA3-GC* and *CAN1-GC*). The mutation rates of the wild-type and *met18Δ* strains are shown in blue and red, respectively. Error bars indicate 95% confidence limits. **(A)** Mutator phenotype of *met18Δ* on *URA3-WT* and *URA3-GC* genes. **(B)** Mutator phenotype of *met18Δ* on *CAN1-WT* and *CAN1-GC* genes.

We further investigated the mutator phenotype associated with *MET18* deletions (*met18Δ*). The strains DKy361 and DKy360 contain the *URA3-GC* and *URA3-WT* alleles, respectively. The mutation rates of *URA3-GC* and *URA3-WT* in the *met18Δ* strains (relative to the rates observed in the isogenic wild-type strains) were elevated 133- and 35-fold, respectively (Table 1). The mutation rate of the *CAN1* gene that had the wild-type base composition (*CAN1-WT*) was elevated about four-fold by the *met18–410C* and about six-fold by the *met18* deletion (Table 1 and Figure 1). Confidence limits on the rates in Table 1 are shown in Supplementary Table S3.

We also examined the effect of the *met18Δ* allele in strains with a *CAN1* variant (*CAN1-GC*) in which the first 804 bps were engineered to have a GC-content of 63.8% and the terminal 966 bp were left unchanged (39% GC) (Supplementary Figure S3). The mutation rate of *CAN1-GC* in the *MET18* wild-type strain was about twice as high as that of the *CAN1-WT* gene (Table 1). The mutation rate of *CAN1-GC* in the *met18Δ* strain was elevated about 15-fold relative to the rate in the *MET18* strain (Table 1). In the *MET18* genetic background, mutations were approximately equally divided between the 5′ 804 bps and the 3′ 966 bps of the *CAN1* gene (42 and 56, respectively) (Supplementary Table S4). In contrast, in the *met18Δ* background, the numbers of mutations in the 5′ and 3′ regions of *CAN1-GC* (51 and 5, respectively) were biased to the 5′ GC-rich interval.

### Mutational spectra in *met18* strains

Both *met18–410C* and *met18Δ* have a unique mutational signature for *URA3-GC* compared to wild-type strains (Supplementary Tables S3 and S5). In the *met18–410C* strain, 38 of 44 (86%) of the *URA3-GC* mutations were deletions (≥5 bp) flanked by short direct repeats (typically, 4–11 bp long), and 61 of 75 (81%) of the mutations in the *met18Δ* strain were of this class. In the wild-type strain, 25% (23 of 91) of the *URA3-GC* mutations were similar deletions (25). Although deletions ≥ 5 bp were not observed for *URA3-WT* in the wild-type strain (25,43) this type of mutation accounted for over 50% of *URA3-WT* mutations in both the *met18–410C* (51 of 79) and *met18Δ* (36 of 63) strains (Supplementary Table S5). This difference represents more than a 1,000-fold increase in the rate of long deletions among *URA3-WT* mutations as a result of the *met18* mutation.

A similar effect of *met18* was observed for the *CAN1* gene. In the wild-type strain with the *CAN1-WT* gene, only 2 of 56 mutations (4%) were deletions ≥5 bp, whereas in the *met18Δ* strain, 41 of 54 mutations (76%) were of this class (Supplementary Table S5). In the hybrid *CAN1-GC*, the wild-type *MET18* strain had 7 deletions ≥5 bp of 101 total mutations (7%), and the *met18Δ* strain had 60 of 61 mutations (98%) of this class (Supplementary Table S5), concentrated in the GC-rich half (Supplementary Table S4). The sequences of all deletions and duplications ≥5 bp for *URA3* and *CAN1* for all strains are shown in Supplementary Table S6.

**Figure 2.**
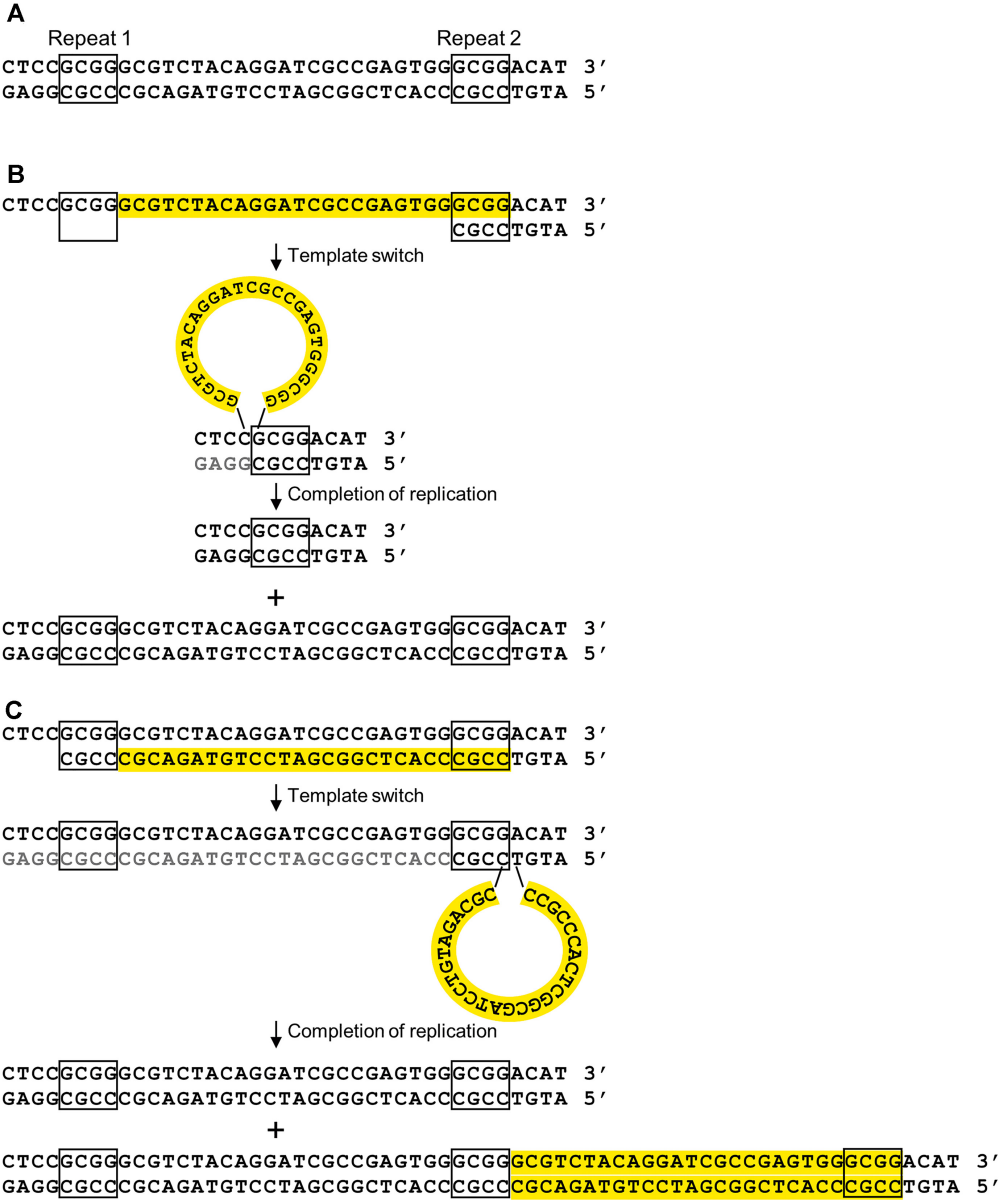
DNA polymerase slippage events resulting in deletions and duplications. The *URA3* gene is likely replicated from *ARS508* located to the left of the gene (70). (**A**) Sequence of the *URA3-GC* gene (bases 315–353) that contains two 4 bp repeats (boxed) that are found at the junctions of deletions and duplications. (**B**) Deletion resulting from slippage of the primer strand during replication between Repeats 1 and 2. The expected products are one chromosome with the original sequence and one with a 23 bp deletion. (**C**) Duplication resulting from replication of Repeats 1 and 2, followed by dissociation of the primer strand from Repeat 2 to Repeat 1, and continued DNA synthesis. The expected products are a chromosome with the original sequence and one with a duplication.

It is likely that the deletions ≥5 bp reflect DNA polymerase slippage. As mentioned previously, mutations in Pol δ substantially elevate this class of mutation. Although non-homologous end-joining might produce similar types of deletions, we showed previously that the rate of deletions of *URA3-GC* in the wild-type strain were not reduced by mutations that eliminate end-joining events (25); in addition, *in vitro* synthesis by DNA polymerase δ can generate similar deletion mutations (44). Although DNA polymerase slippage can result in both deletions (Figure 2A) and duplications (Figure 2B), in the current study, in the *met18* strains, deletions outnumber duplications very substantially, 299 to 4.

In *met18* strains, another common class of mutations in *URA3-GC* was an insertion of the dinucleotide TC between bases C366 and G367 (designated *ura3-GC-366TC*) which was observed in 14% (19/134) of the *URA3-GC* mutations (Supplementary Table S5). The high frequency and context of this mutation argues that it may be a templated mutation rather than random event, resulting in the conversion of a quasi-palindrome (QP) sequence to a perfect palindrome (Supplementary Figure S4A). Similar mutations were first reported in *Escherichia coli* (45). One mechanism to explain this class of mutation (reviewed in (46)) involves two cycles of template switching as shown in Supplementary Figure S4B; by this model, mutations that render DNA polymerase less processive would be expected to elevate the frequency of this class of mutation. An alternative model is that the mutation reflects mismatch repair in a ‘hairpin’ structure formed between the two components of the imperfect palindrome (45). By this mechanism, the frequency of the *ura3-GC-366TC* mutation should be reduced in a strain deficient in mismatch repair. Since there was a two-fold elevation in the frequency of the *ura3-GC-366TC* mutation in the strain with the *met18 mlh1* genotype (Table 1), we prefer the template-switching model.

### Mutations in the Fe–S binding site of Pol3p (the catalytic subunit of Pol δ) mimic the mutagenic effects of the *met18* mutation

Since mutations in *POL3* result in an elevation in deletions flanked by short direct repeats and since the Pol3p contains an iron-sulfur cluster (24), we hypothesized that Pol δ might be the relevant target of the mutagenic effects of *met18*. Four cysteines in Pol3p (C1056, C1059, C1069, C1074) constitute the Fe–S binding domain (19). We examined the phenotypes associated with replacing cysteine with serine in three of these four positions (C1059S, C1069S, C1074S); C1074S is the same substitution previously described as the allele *pol3–13* (22,47).

**Figure 3.**
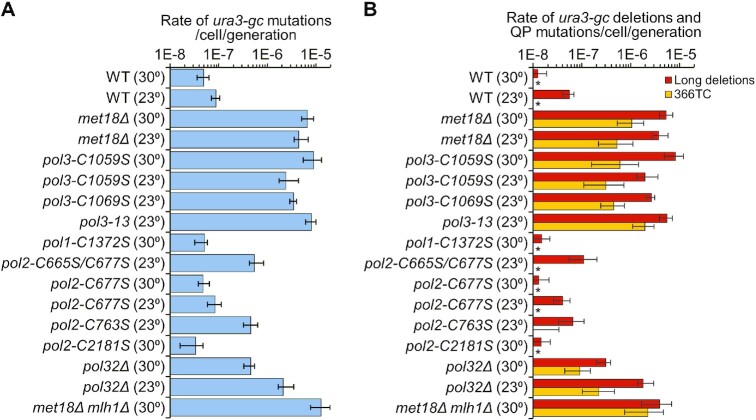
Rates of *URA3-GC* mutations and rates of deletions (≥ 5 bp) and QP mutations in various genetic backgrounds. Error bars indicate 95% confidence limits. Numbers in parentheses show whether the rates were determined in 23°C or 30°C cultures. WT indicates the wild-type strain. (**A**) Rates of *URA3-GC* mutations. (**B**) Rates of deletions and QP (*ura3-GC-366TC*) mutations. As shown in Supplementary Figure S4, the QP mutation is likely the consequence of template switching between two inverted repeats, resulting in formation of a perfect palindrome from a quasi-palindrome. Rates of other types of mutations in each strain are shown in Supplementary Table S3. Asterisks indicate that no events were observed for that class of mutation.

Both the mutation rates and the mutation spectra for *URA3-GC* were very similar in strains with the *pol3-C1059S* mutation or the *met18Δ* mutation (Table 1, Supplementary Tables S3 and S5). The 95% confidence limits overlapped for the total *URA3-GC* mutation rates, and the rates of the two major classes of mutations, long deletions and QP mutations (Supplementary Table S3); for this analysis, we only compared rates and spectra measured at the same temperature. Moreover, by the same statistical test, the mutation rates and spectra (long deletions and single bp substitutions) in *CAN1-WT* were not significantly different in the *pol3-C1059S* and *met18Δ* strains (Table 1, Supplementary Tables S3, and S5). These data are summarized in Figure 3.

Although strains with the *pol3-C1059S* mutation grow normally at 30°C, strains with the *pol3–13* (*pol3-C1074S*) and *pol3-C1069S* mutations grow slowly at this temperature. Consequently, we assayed the mutagenic effects of these mutations in cells grown at room temperature (23°C). As controls, we also assayed the *pol3-C1059S* (*URA3-GC*) and the *met18Δ* (*URA3-GC*) strains at 23°C. There was no significant difference in the rates of mutations in the comparison of the *met18Δ* strain with *pol3–13* or *pol3-C1069S* (Supplementary Table S3; Figure 3). However, the rate of *ura3* mutations in the *pol3–13* strain was significantly elevated (∼2.5-fold) compared to the rates in *pol3-C1059S* and *pol3-C1069S*. Similarly, the rates of both deletions ≥5 bp and QP mutations were significantly elevated in the *pol3–13* strain relative to the rates in the *pol3-C1059S* and *pol3-C1069S* strains (Supplementary Table S3). These results suggest that either there are different effects of different Fe–S-binding-site mutations on Fe–S binding or that that these mutations can also alter the catalytic activity of Pol3p independent of Fe–S binding. For all mutations that disrupted the Fe–S binding site, the predominant class of mutations was deletions ≥ 5 bp flanked by short direct repeats; the *ura3-GC-366TC* mutation was also a prominent class.

Although these results strongly implicate Pol δ as responsible for the mutator phenotype in *met18* cells, we also examined the phenotypes associated with mutating the Fe–S binding sites of Pol ϵ. The Fe–S cluster binding pocket in Pol2p (encoding the catalytic subunit of Pol ϵ) was originally described to include C2164, C2167, C2179 and C2181 (19) but later was amended to include C665, C668, C677, and C763 (48). To test both sites, we constructed three strains with single cysteine substitutions in *POL2* (*pol2-C2181S*, *pol2-C677S*, *pol2-C763S*), and one strain with a double substitution (*pol2-C665S/C677S*). The *pol2-C677S* and *pol2-C2181S* mutations had no significant effect on mutation rates of *URA3-GC* compared to the wild-type strain (overlap of 95% confidence limits on rates measured at 30°C, Supplementary Table S3).

Since strains with the *pol2-C763S* mutation and the double mutant combination *pol2-C665S/C677S* grew very slowly at 30°C, we assayed mutation rates at 23°C. An unanticipated result of this analysis was that the mutation rate of *URA3-GC* in the wild-type strain was elevated about two-fold compared to the rate measured at 30°C, with a 5-fold elevation in large deletions (Table 1). The strains with the *pol2-C763S* and the *pol2-C665S/C677S* had *URA3-GC* mutation rates that were elevated about 5-fold relative to the wild-type rate (Table 1); most of this elevation was a consequence of an increase in the rates of single bp substitutions. In contrast, the strain with the *pol2-C677S* mutation had no significant effects on the rate or spectra of mutations compared to the wild-type strain (Table 1, Supplementary Table S3). This result argues that different cysteines in the binding site have different effects on the binding of the Fe–S cluster or that these mutations have different effects on the fidelity of Pol ϵ. In addition, we cannot rule out the possibility that some of the mutations reflect an indirect effect of the *pol2* mutations. For example, if some of the *pol2* mutations result in an increased role of Pol δ in replicating the leading strand, it is possible that the wild-type Pol δ enzyme makes more errors when replicating the ‘wrong’ strand.

Although the elevation in mutation rates observed for some of the *pol2* mutants was significant, it was much less than the >100-fold increase in rate observed for *URA3-GC* in the *met18Δ* strain or the strains with the mutations in the Fe–S binding site in Pol3p. In addition, deletions ≥5 bp were a much smaller fraction of the total mutations in *URA3-GC* for the *pol2-C763S* and *C665S/C677S* strains (14% and 20%, respectively) than for the *met18Δ* (81%) or the strains with the mutations in the Fe–S binding site in Pol3p (mean of 78%). Taken together, our data suggest that the mutagenesis observed in *met18* strains is a consequence of loss of the Fe–S cluster in Pol3p rather than Pol2p.

### Rates of *URA3* and *CAN1* single-base substitution mutations in *met18* and mutant Pol δ strains

No point mutations were observed in the *URA3-GC* reporter in the *met18* strain and few were found in the Pol δ mutant strains (Supplementary Table S5). It is likely that this result simply reflects the small numbers of sequenced *ura3* mutations and the very large increase in deletions rather than a lack of point mutations. With the *URA3-WT* reporter, we found that the *met18Δ* mutation elevated single-base mutations by about 12-fold relative to the wild-type strain. This rate was only reduced two-fold by deleting *REV3*, encoding the catalytic subunit of the error-prone Pol ζ (Table 1). The *pol3-C1059S* mutation increased the rate of single-base mutations in *URA3-WT* by 18-fold, and the rate of mutations in *CAN1-WT* by about 4-fold (Table 1). These observations indicate that absence of the Fe–S cluster in Pol3p may reduce base selectivity of the holoenzyme.

The elevation in the rate of *CAN1* mutations is similar to that observed previously in *pol3–13* strains which was dependent on Rev3p (47). The interpretation of the effects of Rev3p in our experiments with the *met18* mutation is complicated by the observation that Rev3p contains an Fe–S cluster that is likely inserted by the CIA complex (49).

The locations and the types of single-base substitutions in *URA3* and *CAN1* are shown in Supplementary Table S7. Since the mutation spectra of *met18* strains with the *URA3-GC* reporter gene are heavily dominated by deletions, we restricted our comparisons of types of single-base substitutions to strains containing the *URA3-WT* and *CAN1-WT*. Several interesting patterns were observed. First, the proportion of AT to TA mutations for *URA3-WT* was substantially elevated in *met18* and *pol3-C1059S* strains (mean of 0.43 versus mean of 0.13, respectively; Fisher exact test *P* <0.0001; Supplementary Figure S5A). The same pattern was evident for *CAN1-WT* (Supplementary Figure S5B). Second, the proportions of CG to GC mutations in the *met18* and *pol3-C1059S* strains in *URA3-WT* were elevated relative to wild-type (0.13–0.17, not significant) and an elevation of this substitution was also observed for mutations in *CAN1-WT* (0.16–0.36; *P* = 0.005); an elevated frequency of CG to GC mutations was shown previously to be Pol ζ dependent (50,51). Third, two mutations in *URA3-WT*, T to A at positions 164 and 168, were frequently found in the *met18–410*, *met18Δ*, and *pol3-C1059S* strains (total of 11 such mutations out of a total of 75), but were absent from the wild-type spectrum (0 of 228 mutations) (Supplementary Table S7, *P* <0.0001 by the Fisher exact test).

### Mutation accumulation experiments in the *met18Δ* strain

To determine whether the patterns of mutations observed with *URA3* and *CAN1* were representative of the whole genome, we sequenced the genomes of ten haploid *met18Δ* strains that had been sub-cultured 40 times (about 1000 cell divisions/isolate). The strain used for this analysis (DKy361) was initially haploid, although 3 of the 10 derivatives diploidized during the sub-culturing; such unselected diploidization events have been observed previously in sub-cultured haploids (52,53), and likely reflect the faster growth rate of diploids.

The types of mutations and the genomic location of the mutations are shown in Supplementary Table S8 (details in Supp. Exp. Procedures). Mutations were aligned relative to a wild-type W303–1A reference strain (TAK948; (54)) and the relative rates of various classes of mutations were determined using data from the wild-type strain delta|(-2)|-7B-YUNI300 (34). Compared to the rate of mutations in the wild-type strain, *met18* elevated genomic mutation rates over seven-fold (15 × 10^−10^/bp/cell division) with elevations of the rates of specific types of mutations as follows: single-base substitutions (6-fold), 1 bp deletions (14-fold), 1 bp insertions (3-fold), deletions greater than 5 bp (24-fold), insertions greater than one bp (3-fold), and complex mutations (16-fold). Of the six different types of base substitutions, the most significant alteration (*P* < 0.0001) was a 22-fold elevation in the rate of AT to TA mutations. The relative proportions of the six types of base substitutions are shown in Supplementary Figure S5C.

As observed in the analysis of the *URA3* and *CAN1* genes, we observed mutations that indicated increases in template-primer dissociations, including multi-base deletions and complex mutations (Supplementary Table S8). Most of the multi-base deletions were greater than 5 bp, and were flanked by short homologies, implying that they were generated by polymerase slippage (Figure 2). Of the 11 complex mutations, five perfected palindromic sequences or inverted hairpin loops, implying template switches (Supplementary Figure S4). Four complex alterations appear to reflect gene conversion events between non-allelic repeats (Supplementary Table S8). In summary, the whole-genome analysis of mutations in the *met18Δ* strain recapitulate the patterns observed in the *URA3* and *CAN1* genes.

### The *met18Δ* mutation reduces binding of the Fe–S cluster to Pol3p, resulting in decreased polymerase processivity

**Figure 4.**
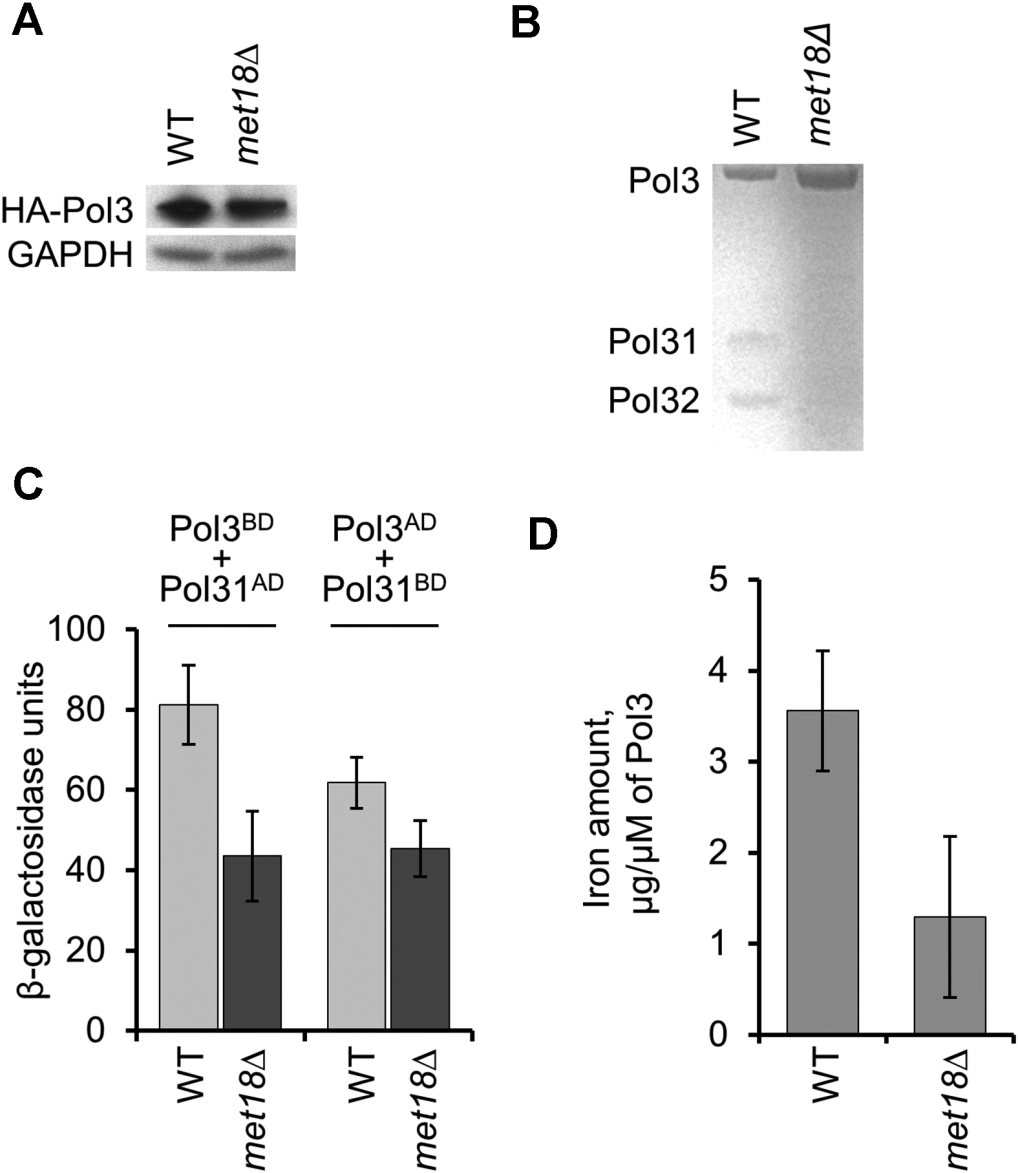
Properties of the Pol3p enzyme isolated from wild-type and *met18* strains. Error bars indicate standard errors of the mean for Figure 4C, and 95% confidence limits for Figure 4D. (**A**) Western blot showing that the amount of Pol3p is similar in wild-type and *met18* strains. An antibody directed against an HA tag was used to monitor the level of HA-tagged Pol3; an antibody specific to glyceraldehyde 3-phosphate dehydrogenase (GAPDH) was used as a control. (**B**) Using a method that conserves the interaction between Pol3p and its subunits Pol31p and Pol32p, we isolated Pol3p and examined its composition by gel electrophoresis. Although the wild-type enzyme consists of three equimolar subunits, the Pol3p isolated from the *met18* strain is missing Pol31p and Pol32p. (**C**) Two-hybrid analysis of the interaction between Pol3p and Pol31p (details in Supp. Experimental Procedures). The level of beta-galactosidase reflects the level of interaction between Pol3p and Pol31p. The Pol3p-Pol31p interaction is significantly reduced in the *met18* strain. (**D**) FPLC-purified Pol3p, examined by inductively coupled plasma mass spectrometry (ICP-MS), has significantly less bound iron in samples isolated from *met18* strains compared to wild-type strains.

Since we have previously shown that low levels of Pol δ elevate the rate of deletions ≥5 bp (15,16), it is possible that the absence of the Fe–S cluster in Pol3p results in an unstable protein. To test this possibility, we used *MET18* and *met18Δ* strains expressing Pol3p with a N-terminal HA tag regulated by the endogenous *POL3* promoter (15). The steady-state levels of Pol3p in these two strains were indistinguishable (Figure 4A).

We extracted and analyzed FPLC-purified Pol3p from yeast cells over-expressing Pol3p and the other subunits of Pol δ (Pol31p and Pol32p) from galactose-inducible promoters. Pol3p purified from the *met18Δ* strain was a single subunit, whereas Pol3p from the *MET18* strain co-purified with accessory subunits Pol31p and Pol32p in approximately equimolar ratios relative to Pol3p (0.8 for Pol31p and 1.1 for Pol32p) (Figure 4B). We also examined the interactions between Pol3p and Pol31p using a two-hybrid analysis. In strains that contained Pol3 fused to a LexA DNA-binding domain (Pol3^BD^) and Pol31 fused to a *GAL4*-activation domain (Pol31^AD^), the level of β-galactosidase synthesized from a (lexA_op_)_8_-lacZ reporter gene was reduced about two-fold (*P* < 0.01) in the *met18* strain relative to the wild-type strain (Figure 4C). A significant (*P* < 0.01) reduction was also observed in strains in which the activation domain and DNA-binding domain were swapped between Pol3 and Pol31 (Pol3^AD^ and Pol31^BD^).

We found that Pol3p isolated from *met18Δ* strains had significantly less iron in comparison to samples from *MET18* strains as detected by the inductively-coupled plasma mass spectrometry (ICP-MS) analysis (Figure 4D). These results argue that, in the absence of Met18p, Pol3p binds the Fe–S cluster less efficiently, leading to a defect in the formation or the stability of its interactions with Pol31p and Pol32p. These observations are in good agreement with the finding that mutations in the Fe–S binding domain of Pol3p prevent binding of iron and abrogate the interaction of Pol3p with Pol31p and Pol32p (19).

**Figure 5.**
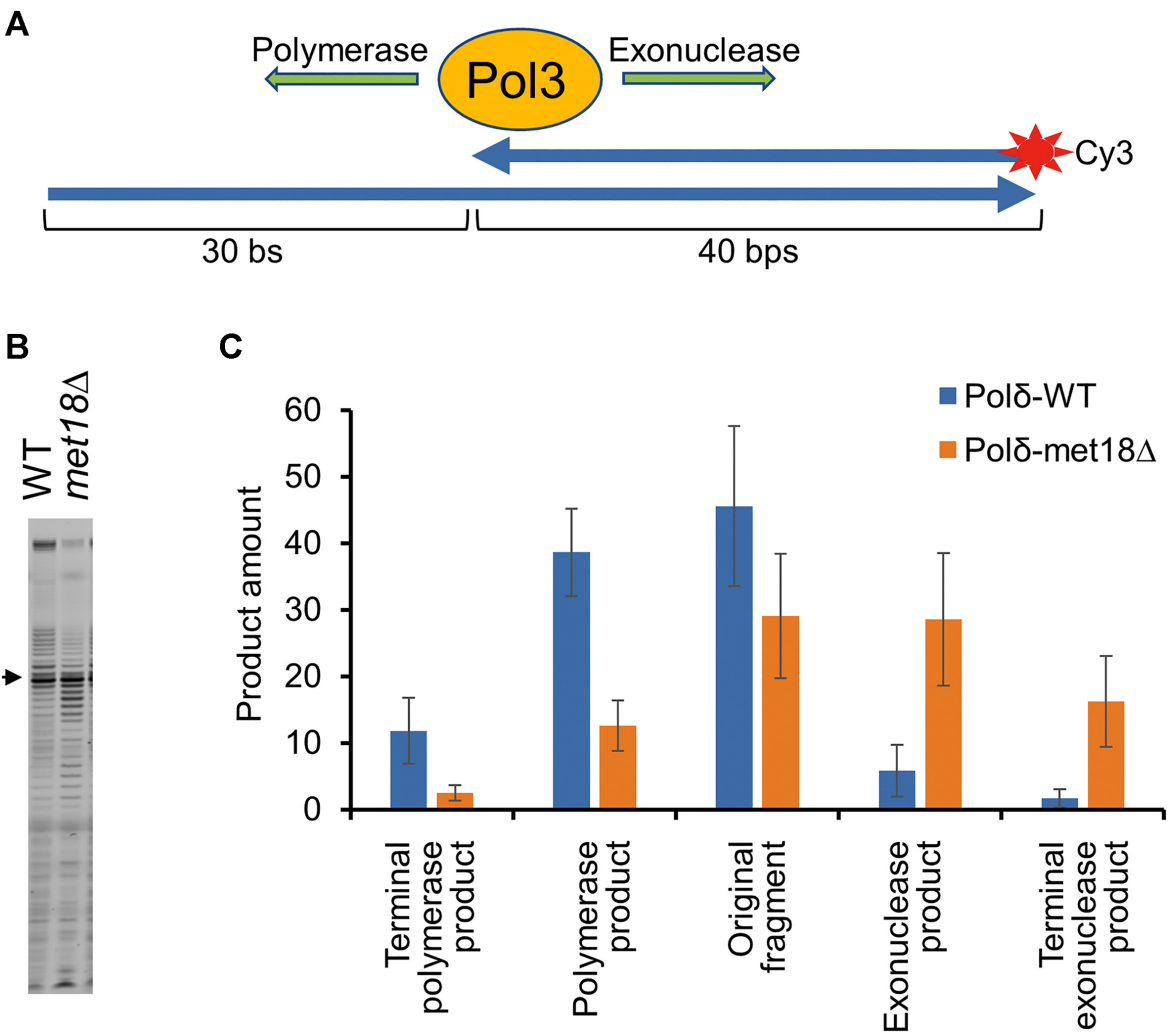
Analysis of *in vitro* DNA synthesis catalyzed by Pol δ isolated from wild-type and *met18* strains. Error bars show standard deviations. (**A**) Schematic of the primed-template DNA synthesis assay used to evaluate the catalytic activity of Pol3p (details in Supp. Expt. Procedures). (**B**) Gel analysis of the Cy3-labeled DNA synthesis products generated as shown in Figure 5A. The arrow shows the position of the 40 bp primer. Products that migrate more slowly than the primer indicate DNA synthesis; products that migrate faster than the primer indicate exonuclease activity. (**C**) Quantitation of the reaction products shown in (B).

We compared the *in vitro* biochemical properties of Pol δ extracted from the *met18Δ* strain (purified as a single Pol3 subunit) and Pol3p from the wild-type strain (which copurified with Pol31p and Pol32p). They did not significantly differ in binding to the DNA template but had different catalytic activities (Figure 5). Relative to the Pol3p/Pol31p/Pol32p complex from the wild-type strain, in the presence of cellular dNTP concentrations, the Pol3p isolated from the *met18Δ* strain exhibited reduced polymerase processivity with a concomitant increase in exonuclease activity. Low polymerase processivity of the enzyme from the *met18Δ* strain provides a mechanistic explanation of the high rates of deletions and other mutations, likely resulting from DNA polymerase slippage and/or template switching.

Although there have been no previous studies of the effect of the *met18* mutation on the enzymatic properties of DNA polymerase δ, (55) examined the activities associated with human DNA polymerase δ with a mutation in a cysteine in the catalytic sub-unit required for Fe–S binding (C1076, equivalent to C1074 for the yeast enzyme). Unlike the yeast enzyme, this mutation in POLD1 resulted in only a slight reduction in its association with the sub-units POLD2, POLD3 and POLD4. It is possible that the POLD4 sub-unit, which is missing in yeast, helps to stabilize the complex. The mutant enzyme had a partial defect in both polymerase and exonuclease activities that could be alleviated by addition of PCNA. In an *in vitro* assay of mutagenesis, the human mutant polymerase had only a small (about 3-fold) elevated rate of mutagenesis, and the frequencies of deletions and insertions were not elevated compared to the frequency of other types of mutations. In summary, although the human holoenzyme lacking the Fe–S cluster has some of the same properties as the yeast enzyme isolated from *met18* strains, there are also a substantial number of differences between our observations and those of (55). These differences could reflect the intrinsic properties of human and yeast enzymes (perhaps related to the presence or absence of POLD4), different amounts of retained Fe–S in mutant DNA polymerases versus polymerases isolated from *met18*, and/or different experimental approaches.

### Mutation rates in *pol32Δ* strains

We also examined the effects of a *POL32* deletion on mutagenesis of *URA3-GC*. Strains with this mutation fail to grow at 13°C and grow poorly at 30°C (13). In assays of mutagenesis in *pol32Δ* strains grown at 30°C, Huang *et al.* (56) found no significant effect on the rate of *CAN1* mutations, but found an increased proportion of deletions. In our genetic background, *pol32Δ* strains grew more slowly than the wild-type strain at 30°C but at approximately the same rate at 23°C. In our study, the rates of *URA3-GC* mutations were elevated at both 30°C and 23°C (Table 1). Compared to the wild-type rate at 30°C, the rate in the *pol32Δ* strain was elevated by 9-fold at 30°C and by 42-fold at 23°C. For comparison, the rates of *URA3-GC* mutations in the *met18Δ* strain and the strains with mutations in the binding site of the Fe–S cluster (for example, *pol3-C1059S*) were elevated 133- and 181-fold, respectively, at 30°C. These results suggest that the elevated rate of mutagenesis observed in the *pol3* and *met18* strains are, at least in part, independent of the loss of Pol32p from Pol δ.

The types of mutations observed in the *pol32Δ* strains were similar to those observed in the *met18* and *pol3* strains (Supplementary Table S5). The percentages of the total *URA3-GC* mutations that were deletions ≥ 5 bp in strains grown at 30°C were: 25% (wild-type), 81% (*met18-Δ*), 92% (*pol3-C1059S*) and 68% (*pol32-Δ*). In addition, the quasi-palindrome mutation *ura3-GC-366TC* accounted for more than 10% of the *URA3-GC* mutations in the *pol32Δ* strain grown at either 23 or 30°C (Supplementary Table S5). These results suggest that the mutagenic effects of the *met18* mutation could be a consequence of two factors, the loss of interaction with the Pol32p subunit and a Fe–S-dependent loss of processivity independent of that interaction. This issue will be discussed further below.

## DISCUSSION

The main conclusions of our study are: 1. The *met18* mutation has a mutator phenotype that preferentially affects genes with high-GC contents. 2. Strains with the *met18* mutation or with mutations in Pol δ that prevent binding of the Fe–S cluster have similar rates and spectra of mutations, arguing that the mutagenic effects of *met18* are a consequence of loss or destabilization of the Fe–S cluster in Pol δ. 3. The variant form of Pol δ that lacks the Fe–S cluster or has a destabilized Fe–S cluster has an elevated rate of DNA polymerase slippage/template switching *in vivo* and decreased processivity *in vitro*. We suggest that many of these observations are consistent with the hypothesis that the binding of PCNA to Pol δ is regulated by the Fe–S cluster.

### Identification of *met18* as a mutator and caveats concerning mutator screens

Our analysis demonstrates that the *met18* mutation elevates mutation rates in the *URA3-GC* gene more than 100-fold, but has a smaller effect (35-fold) on the *URA3-WT* gene that has an average GC content. The mutator phenotype by whole-genome analysis is only 7-fold. We do not know the explanation for the higher mutation rate of *URA3-GC* gene relative to *URA3-WT* in the *met18* strain, which is also observed for these reporters in the wild-type strain (25). It is possible that the yeast replicative enzymes are generally less accurate on high-GC templates. Alternatively, the GC content may affect mutagenesis less directly. For example, the tendency of high-GC genes to form stable R-loops (57) may reduce the processivity of the polymerases.

Although there have been successful screens to identify mutators in yeast (for example, (58)), the *met18* gene was identified as a mutator in only one such screen (59). The limitations of screening ‘knock-out’ collections for mutators involve a number of factors. First, some mutator mutations may be in essential genes and will be missing from a knock-out collection. Second, often the screens are done using a single reporter gene and, as illustrated by our analysis, mutators may have sequence specificity. Third, for at least some mutators, the temperature during cell growth affects the magnitude of the mutator effect. The *pol32Δ* mutation has a five-fold greater effect at 23°C than 30°C (Table 1). There is no simple solution to these issues except to acknowledge that high-throughput screens are limited to detecting a sub-set of mutations in a sub-set of genes.

### Identification of Pol3p as the target for the mutagenic effects of the *met18* mutation

Proteins with Fe–S clusters and the protein complexes necessary to insert these clusters into their target proteins are found in organisms from prokaryotes to humans. In yeast, the proteins involved in the maturation and assembly of Fe–S cluster are essential except for Met18p (60). There are numerous yeast proteins with Fe–S clusters including the catalytic subunits of the Pols δ, ϵ, and ζ, primase, and the helicases Chl1p, Dna2p, and Rad3p (61).

As discussed previously, both the rates and the spectra of *URA3-GC* mutations in both the *met18* strains and the *pol3* strains with mutations in the Fe–S binding cysteines of Pol δ are very similar with most (>88%) of the mutations being deletions or quasi-palindrome insertions. The proportion of these types of *URA3-GC* mutations is substantially lower in the wild-type strain (25%) and in strains with mutations in the Fe–S binding sites of Pol2p (average of 32%) (Supplementary Table S5), implicating Pol3p as the relevant target gene. A related argument implicating Pol δ as the relevant target of mutagenesis in the *met18* strain is that there is a significant increase in the frequency of AT to TA single-base substitutions in both the *met18* strain and in strains with the *pol3-C1059S* mutation (Supplementary Figure S5). Although the similar mutation rates and mutation spectra observed in the *met18* strain and the strains with *pol3* mutations in the Fe–S binding site argue that Pol3 is the most relevant target of the mutagenic effects of *met18*, we cannot exclude the possibility that other Fe–S-containing proteins involved in DNA repair or DNA replication might also contribute to phenotype. Analysis of the mutagenic effects of Fe–S binding site mutations in such proteins would be useful in determining whether these proteins contribute to the mutagenic effects observed in the *met18* strain.

In Figure 3, the DNA polymerase slippage events that generate the deletions and duplication are shown to occur on the lagging strand since the altered Pol δ is responsible for lagging strand replication. However, we have no direct evidence relevant to whether the slippage events occur on the leading or lagging strands. In contrast, because the left and right repeats of the quasi-palindromes are not identical, based on which of these repeats acquires the mutation, we can predict whether the template switching to produce the *ura3-GC-366TC* mutation is initiated on the leading or lagging strand (Supplementary Figure S4). This analysis suggests that this mutation was initiated by a template switch on the leading strand, consistent with previous studies in *E. coli* (46). One explanation for why these QP events are elevated by mutations affecting Pol δ is that this class of mutations requires two template switches, one of which occurs on the lagging strand.

In general, our findings about the mutant phenotypes associated with *met18* for the *URA3* and *CAN1* genes are supported by the whole-genome analysis of the *met18Δ* strain (Supplementary Table S8). One interesting difference is that the proportions of *URA3-WT* and *CAN1-WT* mutations that were deletions ≥5 bp in the *met18Δ* strains (mean of 0.67) were greater than observed by whole-genome analysis (0.07; Supplementary Tables S5 and S8). The likely explanation of this difference is simply that the whole-genome sequencing detects all mutations whereas our analysis of the *URA3* and *CAN1* genes identifies only those mutations that inactivate the encoded proteins. Lang and Murray (43) showed that only 10% of single-base mutations inactivated *URA3* or *CAN1*. Since most deletions will be inactivating, our analysis of *URA3* and *CAN1* over-estimates the fraction of deletion mutations in both wild-type and *met18* strains. The conclusion that the *met18* mutation elevates deletions with respect to the wild-type strain, however, is not affected by this consideration.

In our strains (based on the data of McGuffee *et al.* (62)), for both *URA3* and *CAN1*, transcription and replication fork movement are co-directional. We examined this issue for deletions ≥ 5 bp in the whole-genome analysis. Of the 12 deletions that were within genes, in 10 of these genes, we could determine the origin that likely was responsible for its replication (62). In 5 of the 10 genes, transcription and fork movement were in opposite directions and in five of the genes, transcription and fork movement were in the same direction. Although this dataset is small, we conclude that deletion formation does not occur with a strong preference for genes in which there is a collision between the replication fork and the transcription machinery.

### Possible mechanism for the high rate of deletions in *met18* strains

One model consistent with our observations is that the strong mutagenesis by *met18* on high-GC templates is a consequence of a diminished interaction between PCNA and Pol δ in the *met18* strain. This hypothesis is based on several observations. First, PCNA increases the processivity of Pol δ (63), and certain mutations in *POL30* (encoding yeast PCNA) elevate the frequency of deletions flanked by short direct repeats (64,65). Second, it is possible that PCNA interacts with Pol δ through multiple binding sites. Acharya *et al.* (66) concluded that elimination of putative PCNA-interacting protein (PIP) motifs on Pol3p, Pol32p, and Pol31 reduced processive *in vitro* replication, and PIP mutations in Pol32p substantially reduced interactions with PCNA in overlay blots (67). However, structural studies suggest that the Pol31p is unlikely to contact PCNA, although an interaction between Pol32p and PCNA is not excluded by such studies (68,69). One model is that replication by Pol δ is initiated in *met18* strains by Pol3p, Pol32p, and Pol31p but, in the absence of the Fe–S cluster in Pol3p, this complex frequently breaks down, resulting in loss of the Pol31p and Pol32p subunits at the replication fork, reduced interaction with PCNA, and elevated levels of non-processive synthesis.

Although this model explains most of the observed results, it does not explain why the Fe–S cluster is required for stabilizing the Pol3p–Pol32p–Pol31p complex. Further, the temperature-dependence of the mutagenic effect of the *pol32* mutation is not explained. It is possible that Pol δ is intrinsically more processive at 30°C than 23°C or that PCNA–Pol31p–Pol3p interactions occur more efficiently at the higher temperature. Lastly, we cannot exclude the possibility that loss of the Fe–S cluster from Pol3p also reduces the processivity of Pol δ by a mechanism related to its redox role (21).

## DATA AVAILABILITY

Whole genome sequencing data have been deposited in the Sequence Read Archive database (BioProject ID: PRJNA703018).

*CAN1-GC* sequence can be retrieved using GenBank accession number MW719075.

## Supplementary Material

gkab371_Supplemental_Files
