# Supplementary material for: The fidelity of DNA replication, particularly on GC-rich templates, is reduced by defects of the Fe–S cluster in DNA polymerase δ
Source: Nucleic Acids Res. 2021 May 21;49(10):5623–36. doi: 10.1093/nar/gkab371 (PMC8191807; doi:10.1093/nar/gkab371)
Supplement: gkab371_Supplemental_Files [file gkab371_supplemental_files.zip › Supplemental Figures, Tables S4-S5, and Figure Legends.pdf]

Supplemental Figures

Figure S1. Depiction of mutant screen. A haploid strain with two reporter genes (*URA3-GC* (63% GC) and *CAN1-WT* (41% GC)) was mutagenized with ultraviolet light (UV) and plated on non-selective medium (YPD). We replica-plated the resulting colonies (shown as yellow ovals) to media containing either canavanine (Can) or 5-fluoro-orotate (5-FOA). We isolated for further testing those colonies (example shown by solid blue arrow) that had multiple 5-FOA^R papillae (shown as small yellow ovals) and few or no Can^R papillae. We also performed the same procedure on cells that were not treated with UV to determine how frequently 5-FOA^R or Can^R papillae appeared in unmutagenized cells.

Figure S2. Mapping of a mutator gene using microarrays. We crossed the haploid with the mutator phenotype (DKy240) to a wild-type strain (MD702) with about 50,000 single-nucleotide polymorphisms distinguishing the two haploid parental strains (isogenic with W303-1A and YJM789) (1). The resulting diploid (DKy359) was sporulated, and we identified spores that had a high mutation rate for *URA3-GC*. Genomic samples from ten such spores were isolated and combined in pairs for microarray analysis.

A. Microarray analysis of five pairs of spores with the mutator phenotype. The red line indicates hybridization of the spore DNA to W303-1A-specific SNPs and the blue line shows hybridization to YJM789-specific SNPs. The Y-axis indicates the normalized hybridization level; a level of 1 indicates that one of the spores in a pair had DNA sequences derived from W303-1A and the other had sequences derived from YJM789. The only region of DNA that was associated with the W303-1A strain and was shared in all five spore pairs was the region between Saccharomyces Genome Database (SGD)

coordinates 101815 and 122608 (X-axis) on chromosome IX (bracketed by dotted lines).

B. Genetic features located in the region of interest. These features are shown using the standard panels of the JBrowse feature of the Saccharomyces Genome Database (yeastgenome.org). Sequencing of the individual genes in this region confirmed that the relevant mutation was in *MET18.*

Figure S3. Sequence of *CAN1-GC*. The first 801 bp of *CAN1* (in boldface) were modified by changing A-T base pairs to G-C base pairs without changing the encoded amino acids; the total length of the GC-rich half of *CAN1* (63.8% GC) was 804 bp. The terminal 966 bp are identical to the terminal bases of *CAN1* in the Saccharomyces Genome Database, and have a 39.1% GC content.

Figure S4. A common quasi-palindrome (QP) mutation produced in *URA3-GC* in *met18*, *pol3-C1059S*, *pol3-C1069S, pol3-13* and *pol32Δ* strains.

A. A comparison of the wild-type (top) and mutant (bottom) sequences. The wild-type strain has a quasi-palindrome shown in green and yellow; the green sequence has a two bp insertion relative to the yellow sequence in the wild-type *URA3-GC* strain. This insertion is put into the yellow segment in the *ura3-GC-366TC* mutant gene.

B. Template-switching events that give rise to the QP mutation. The polarity of the mutation requires that the event be initiated by a template switch on the leading strand, followed by a template switch from the lagging strand.

Figure S5. Proportions of different classes of single-base substitutions in wild-type (WT) and *met18* strains, and in a strain with a mutation in the Fe-S-binding domain of Pol δ.

The designation "Σ" represents the sum of the mutations from the *met18-410C*, *met18Δ*, and *pol3-C1059S* strains.

A. Analysis of *URA3-WT* mutations.

B. Analysis of *CAN1-WT* mutations.

C. Analysis of single-base substitutions in wild-type and *met18Δ* strains from mutation-accumulation experiments. The data for the wild-type strain were reported in (2).

Supplemental Tables

Table S1. Strain constructions.

In this table, we show the names, mating types, relevant genotypes, and brief descriptions of strain constructions for all haploid and diploid strains used in the study. Almost all of the strains are isogenic with W303-1A (3,4) except for changes introduced by transformation. The original genotype of W303-1A is: *MAT*a *ade2-1 can1-100 his3-11,15 leu2-3,112 trp1-1 ura3-1*. Although the original version of W303-1A also had the *rad5-G535R* mutation, this mutation is removed in all of our isolates except where noted. In the table, haploid strains that are isogenic to W303-1A are designated "W" in Column C, and isogenic diploids are designated "WxW." The strains used for the isolation of DNA polymerase d are in the BJ2168 background, and are designated "B" in Column C. Strains to examine the level of DNA polymerase d were from the JED213-30 background, and are designated with a "J." Strains used for mapping by SNP-specific microarrays are derived from YJM789 or isogenic strains (5) and are lapeled "Y." Lastly, strains used for two-hybrid analysis are derived from L40 (6) and are indicated by "L".

Most of the strains with mutant DNA polymerase genes were constructed by two-step transplacement in which the mutant gene was inserted into the *URA3*-containing vectors pRS406 or p173 (described in Table S2-1). In the first step, the plasmid is integrated adjacent to the wild-type gene by selecting for Ura^+ transformants. In the second step, we isolate derivatives that have lost the *URA3* insertion by selecting for 5-FOA-resistance. We then sequenced the relevant polymerase gene of the strain to confirm the correct substitution. The plasmid pCORE-UH contains the selectable markers *URA3* (from K. lactis) and *hygR*, and was used to make changes to the *CAN1* locus using the procedure of *delitto perfetto* (7).

Table S2. Plasmids and primers used in strain constructions.

Table S2-1. Plasmids used in the study.

Table S2-2. Oligonucleotide primers used in the study.

Table S3. Rates (with 95% confidence limits) and types of *URA3* and *CAN1* mutations in wild-type and *met18* strains, and in strains with DNA polymerase mutations.

Table S4. Location of mutations in 5' region (1-804) and 3' regions (805-1636 or 805-1770) of *CAN1-WT* and *CAN1-GC*.

Table S5. Numbers of mutations of various classes in *URA3* and *CAN1* in wild-type and *met18* strains, and in strains with DNA polymerase mutations.

Table S6. Sequences of in/dels ≥5 bp in *URA3* and *CAN1* in wild-type, *met18* strains, and in strains with DNA polymerase mutations.

Columns A and B show the reporter gene and the relevant genotype, respectively. Column C shows the temperature at which cells were cultured for the experiment. Column D indicates whether the mutation was a deletion or a duplication.

Columns E and F show the left and right SGD coordinates of the deletion or duplication, and Column G shows the size of the deletion or duplication. The sequences of the repeats bordering the deletion or duplication are shown in Columns H and I. The prime sign indicates borders of the events. Perfectly matched bases within repeats are shown in upper case letters, mismatched bases are shown in lower case letters. The following criteria were used to determine if repeats flanking the events included mismatches: i) one base mismatch was allowed if followed by at least two perfectly matched bases; ii) if two alternative repeats could be produced when applying the first rule, then the longest repeat was preferred; iii) if two alternative repeats had the same length, then the GC-rich repeat was preferred. Only one event in *URA3-GC* in *pol2-C665S,C677S* background could not be resolved using these rules and both alternatives were presented for this mutation. Column K shows the number of independent mutations in a given strain with the identical deletion or duplication.

Table S7. Locations of single-base substitutions and single-base in/dels in *URA3* and *CAN1* genes in wild-type, *met18* strains, and in strains with DNA polymerase mutations.

If a mutant allele had two or more mutations separated by 12 base pairs or less, such mutations were put in the "complex" class; these mutations are outline by thick black lines. In the *pol3-C1059S* strain with the *URA3-WT* reporter gene, we observed one mutant with an insertion of a delta or Ty element with a target 5 bp duplication of AAGCT (bases 8-12 of the *URA3* gene).

Table S8. Genomic mutations derived from mutation-accumulation experiments in a *met18Δ* strain (DKy361).

Table S8-1. Single-base substitutions.

Table S8-2. Deletions or insertions occurring within or adjacent to microsatellites.

Table S8-3. Deletions or insertions ≥ 5 bp flanked by short direct repeats.

Table S8-4. Complex mutations.

Supplemental References

1. St Charles, J., Hazkani-Covo, E., Yin, Y., Andersen, S.L., Dietrich, F.S., Greenwell, P.W., Malc, E., Mieczkowski, P. and Petes, T.D. (2012) High-resolution genome-wide analysis of irradiated (UV and gamma-rays) diploid yeast cells reveals a high frequency of genomic loss of heterozygosity (LOH) events. *Genetics*, **190**, 1267-1284.
2. Williams, J.S., Lujan, S.A., Zhou, Z.X., Burkholder, A.B., Clark, A.B., Fargo, D.C. and Kunkel, T.A. (2019) Genome-wide mutagenesis resulting from topoisomerase 1-processing of unrepaired ribonucleotides in DNA. *DNA Repair (Amst)*, **84**, 102641.
3. Thomas, B.J. and Rothstein, R. (1989) Elevated recombination rates in transcriptionally active DNA. *Cell*, **56**, 619-630.
4. Zhao, X., Muller, E.G. and Rothstein, R. (1998) A suppressor of two essential checkpoint genes identifies a novel protein that negatively affects dNTP pools. *Mol Cell*, **2**, 329-340.

5. St Charles, J. and Petes, T.D. (2013) High-resolution mapping of spontaneous mitotic recombination hotspots on the 1.1 Mb arm of yeast chromosome IV. *PLoS Genet*, **9**, e1003434.

6. Gerik, K.J., Li, X., Pautz, A. and Burgers, P.M. (1998) Characterization of the two small subunits of *Saccharomyces cerevisiae* DNA polymerase δ. *J Biol Chem*, **273**, 19747-19755.

7. Storici, F. and Resnick, M.A. (2003) *Delitto perfetto* targeted mutagenesis in yeast with oligonucleotides. *Genet Eng (N Y)*, **25**, 189-207.

8. Kokoska, R.J., Stefanovic, L., DeMai, J. and Petes, T.D. (2000) Increased rates of genomic deletions generated by mutations in the yeast gene encoding DNA polymerase δ or by decreases in the cellular levels of DNA polymerase δ. *Mol Cell Biol*, **20**, 7490-7504.

9. Kiktev, D.A., Sheng, Z., Lobachev, K.S. and Petes, T.D. (2018) GC content elevates mutation and recombination rates in the yeast *Saccharomyces cerevisiae*. *Proc Natl Acad Sci U S A*, **115**, E7109-E7118.

10. Goldstein, A.L. and McCusker, J.H. (1999) Three new dominant drug resistance cassettes for gene disruption in *Saccharomyces cerevisiae*. *Yeast*, **15**, 1541-1553.

11. Storici, F. and Resnick, M.A. (2006) The *delitto perfetto* approach to *in vivo* site-directed mutagenesis and chromosome rearrangements with synthetic oligonucleotides in yeast. *Methods Enzymol*, **409**, 329-345.

12. Sikorski, R.S. and Hieter, P. (1989) A system of shuttle vectors and yeast host strains designed for efficient manipulation of DNA in *Saccharomyces cerevisiae*. *Genetics*, **122**, 19-27.

13. Stepchenkova, E.I., Tarakhovskaya, E.R., Siebler, H.M. and Pavlov, Y.I. (2017) Defect of Fe-S cluster binding by DNA polymerase δ in yeast suppresses UV-induced mutagenesis, but enhances DNA polymerase ζ - dependent spontaneous mutagenesis. *DNA Repair (Amst)*, **49**, 60-69.

14. Norrander, J., Kempe, T. and Messing, J. (1983) Construction of improved M13 vectors using oligodeoxynucleotide-directed mutagenesis. *Gene*, **26**, 101-106.

15. Kirchner, J.M., Tran, H. and Resnick, M.A. (2000) A DNA polymerase ε mutant that specifically causes +1 frameshift mutations within homonucleotide runs in yeast. *Genetics*, **155**, 1623-1632.

16. Fortune, J.M., Stith, C.M., Kissling, G.E., Burgers, P.M. and Kunkel, T.A. (2006) RPA and PCNA suppress formation of large deletion errors by yeast DNA polymerase δ. *Nucleic Acids Res*, **34**, 4335-4341.

Figure S1

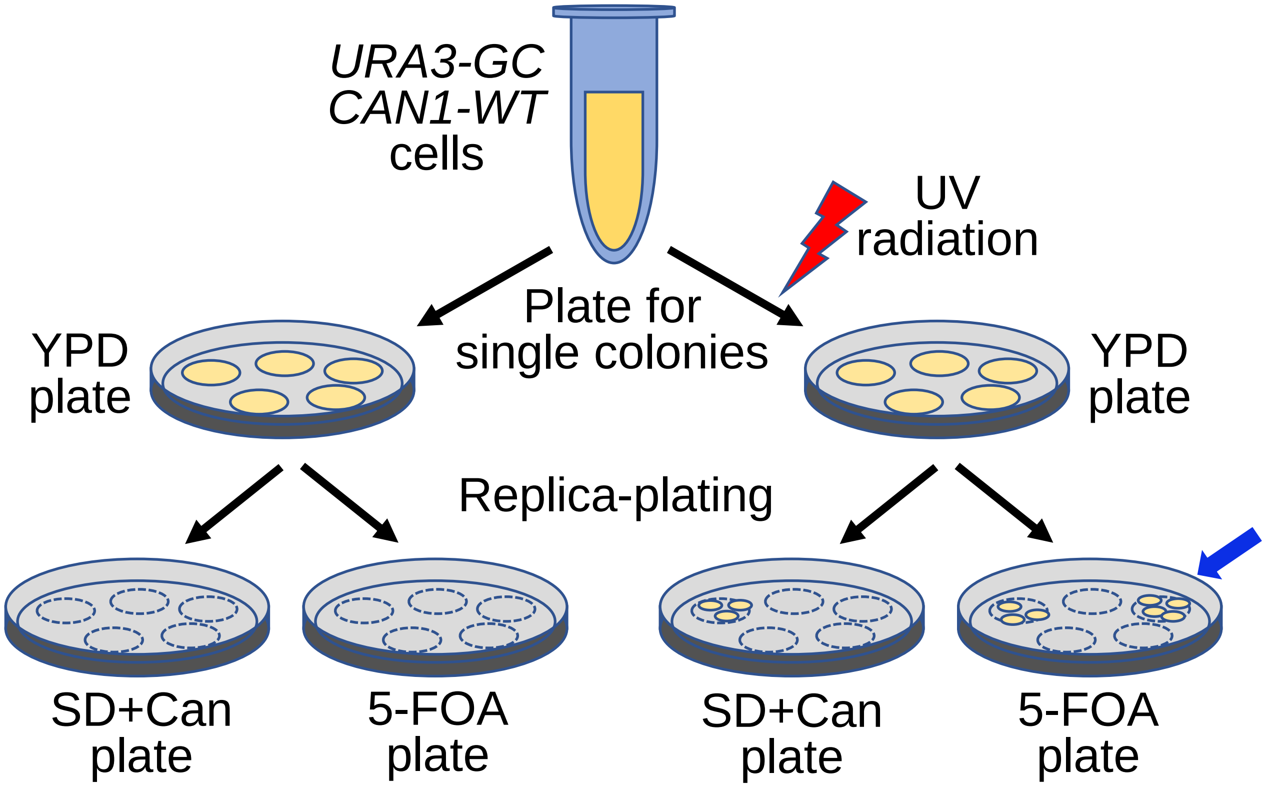
URA3-GC
CAN1-WT
cells
UV
radiation
Plate for
single colonies
YPD
plate
YPD
plate
Replica-plating
SD+Can
plate
5-FOA
plate
SD+Can
plate
5-FOA
plate

Figure S2

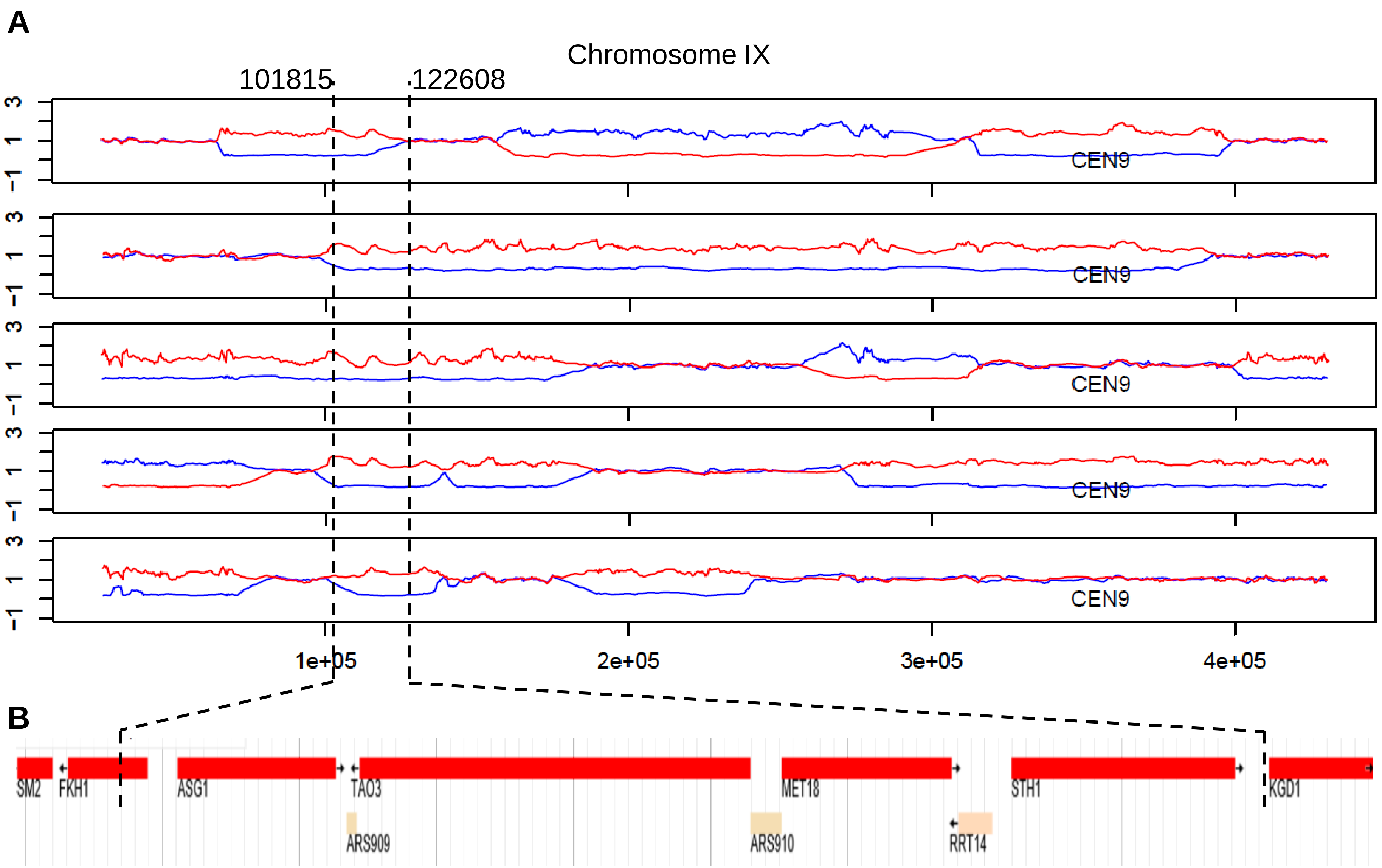
A
Chromosome IX
101815
122608
3
1
−1
CEN9
1e+05
2e+05
3e+05
4e+05
B
SM2
FKH1
ASG1
TAO3
MET18
STH1
KGD1
ARS909
ARS910
RRT14

Figure S3

ATG ACC AAC TCC AAG GAG GAC GCC GAC ATC GAG GAG AAG CAC ATG TAC AAC
GAG CCG GTC ACC ACC CTC TTC CAC GAC GTC GAG GCC TCC CAG ACC CAC CAC
AGG CGT GGG TCC ATC CCC CTG AAG GAC GAG AAG AGC AAG GAG CTG TAC CCG
CTG CGC TCC TTC CCG ACG AGG GTC AAC GGC GAG GAC ACG TTC TCC ATG GAG
GAC GGC ATC GGC GAC GAG GAC GAG GGC GAG GTC CAG AAC GCC GAG GTG AAG
AGG GAG CTG AAG CAG AGG CAC ATC GGC ATG ATC GCC CTG GGC GGC ACC ATC
GGC ACC GGC CTG TTC ATC GGC TTG TCC ACC CCC CTG ACC AAC GCC GGC CCC
GTG GGC GCC CTG ATC TCC TAC TTG TTC ATG GGC TCC TTG GCC TAC TCC GTC
ACG CAG TCC CTG GGC GAG ATG GCC ACC TTC ATC CCC GTC ACC TCC TCC TTC
ACC GTG TTC TCC CAG AGG TTC CTG TCC CCG GCC TTC GGC GCG GCC AAC GGC
TAC ATG TAC TGG TTC TCC TGG GCC ATC ACC TTC GCC CTG GAG CTG AGC GTG
GTC GGC CAG GTC ATC CAG TTC TGG ACG TAC AAG GTC CCG CTG GCG GCC TGG
ATC AGC ATC TTC TGG GTC ATC ATC ACC ATC ATG AAC CTG TTC CCC GTC AAG
TAC TAC GGC GAG TTC GAG TTC TGG GTC GCC TCC ATC AAG GTC CTG GCC ATC
ATC GGG TTC CTG ATC TAC TGC TTC TGC ATG GTC TGC GGC GCC GGG GTC ACC
GGC CCG GTC GGC TTC CGC TAC TGG AGG AAC CCC GGC GCC TGG GGT CCA GGT
ATA ATA TCT AAG GAT AAA AAC GAA GGG AGG TTC TTA GGT TGG GTT TCC TCT
TTG ATT AAC GCT GCC TTC ACA TTT CAA GGT ACT GAA CTA GTT GGT ATC ACT
GCT GGT GAA GCT GCA AAC CCC AGA AAA TCC GTT CCA AGA GCC ATC AAA AAA
GTT GTT TTC CGT ATC TTA ACC TTC TAC ATT GGC TCT CTA TTA TTC ATT GGA
CTT TTA GTT CCA TAC AAT GAC CCT AAA CTA ACA CAA TCT ACT TCC TAC GTT
TCT ACT TCT CCC TTT ATT ATT GCT ATT GAG AAC TCT GGT ACA AAG GTT TTG
CCA CAT ATC TTC AAC GCT GTT ATC TTA ACA ACC ATT ATT TCT GCC GCA AAT
TCA AAT ATT TAC GTT GGT TCC CGT ATT TTA TTT GGT CTA TCA AAG AAC AAG
TTG GCT CCT AAA TTC CTG TCA AGG ACC ACC AAA GGT GGT GTT CCA TAC ATT
GCA GTT TTC GTT ACT GCT GCA TTT GGC GCT TTG GCT TAC ATG GAG ACA TCT
ACT GGT GGT GAC AAA GTT TTC GAA TGG CTA TTA AAT ATC ACT GGT GTT GCA
GGC TTT TTT GCA TGG TTA TTT ATC TCA ATC TCG CAC ATC AGA TTT ATG CAA
GCT TTG AAA TAC CGT GGC ATC TCT CGT GAC GAG TTA CCA TTT AAA GCT AAA
TTA ATG CCC GGC TTG GCT TAT TAT GCG GCC ACA TTT ATG ACG ATC ATT ATC
ATT ATT CAA GGT TTC ACG GCT TTT GCA CCA AAA TTC AAT GGT GTT AGC TTT
GCT GCC GCC TAT ATC TCT ATT TTC CTG TTC TTA GCT GTT TGG ATC TTA TTT
CAA TGC ATA TTC AGA TGC AGA TTT ATT TGG AAG ATT GGA GAT GTC GAC ATC
GAT TCC GAT AGA AGA GAC ATT GAG GCA ATT GTA TGG GAA GAT CAT GAA CCA
AAG ACT TTT TGG GAC AAA TTT TGG AAT GTT GTA GCA TAG

Figure S4

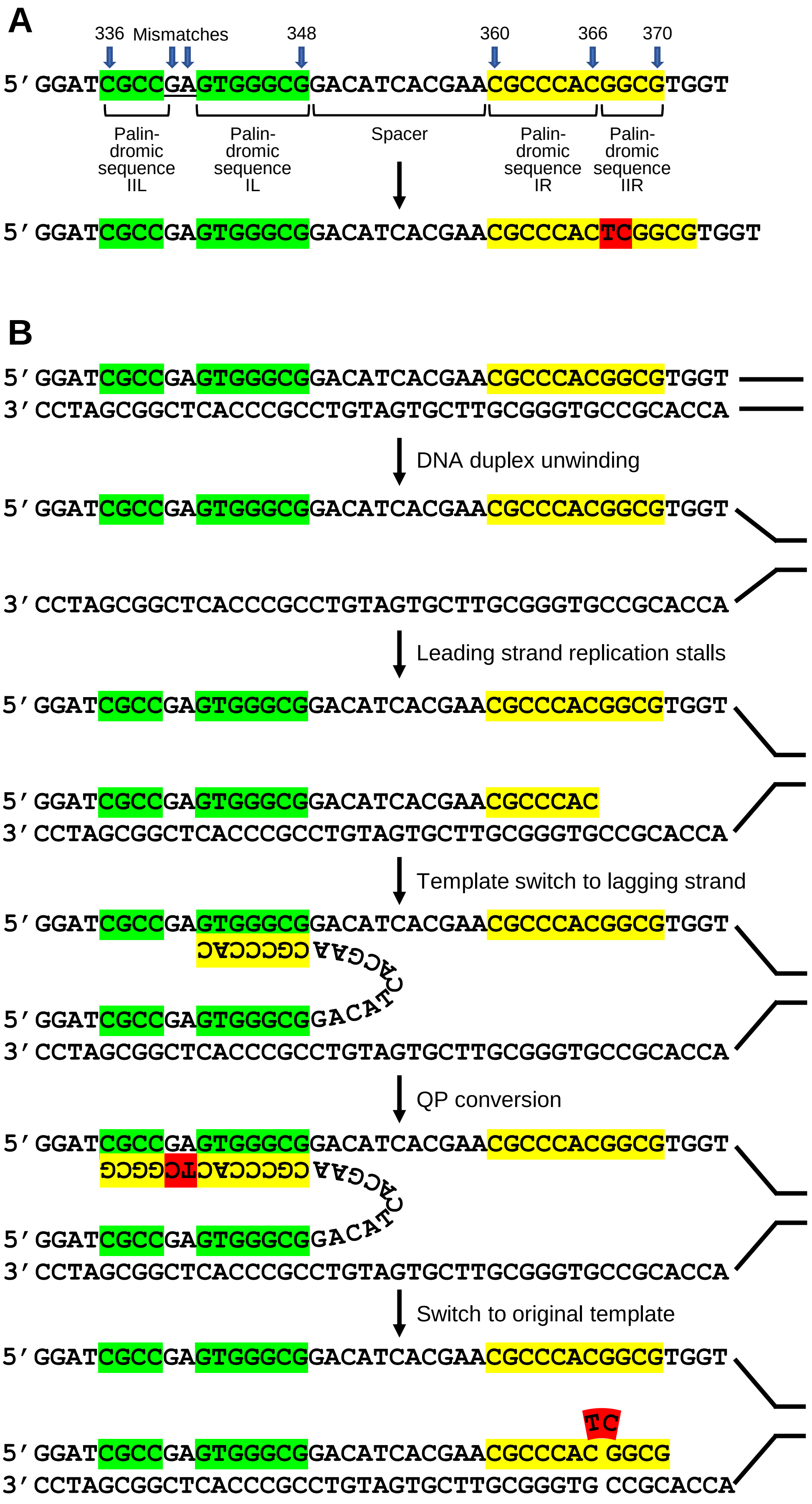
A
336 Mismatches 348 360 366 370
5′GGATCGCCGAGTGGGCGGACATCACGAACGCCCACGGCGTGGT
Palin-dromic sequence IIL
Palin-dromic sequence IL
Spacer
Palin-dromic sequence IR
Palin-dromic sequence IIR
5′GGATCGCCGAGTGGGCGGACATCACGAACGCCCACTCGGCGTGGT
B
5′GGATCGCCGAGTGGGCGGACATCACGAACGCCCACGGCGTGGT
3′CCTAGCGGCTCACCCGCCTGTAGTGCTTGCGGGTGCCGCACCA
DNA duplex unwinding
Leading strand replication stalls
Template switch to lagging strand
QP conversion
Switch to original template

Figure S5

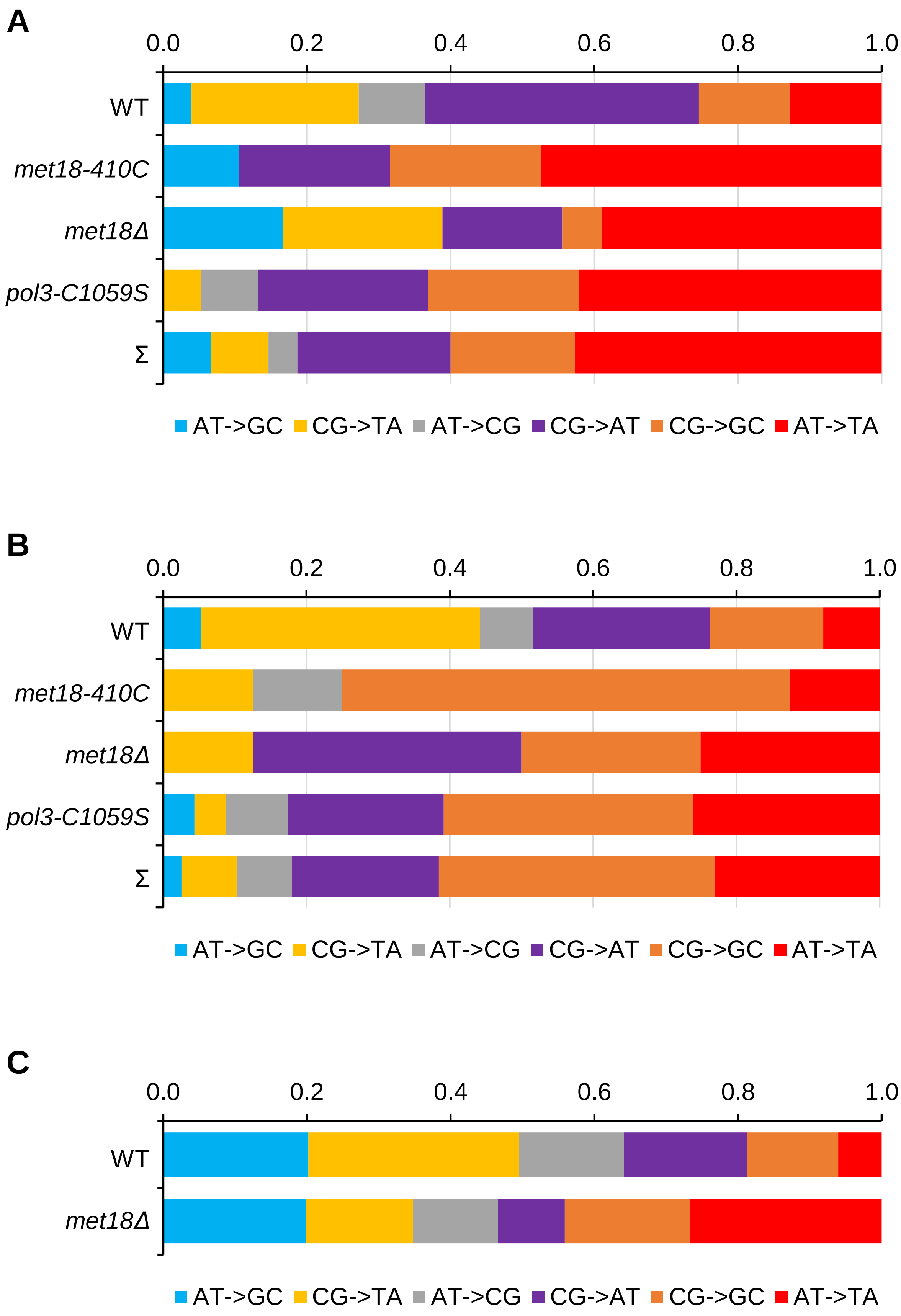
A
0.0
0.2
0.4
0.6
0.8
1.0
WT
met18-410C
met18Δ
pol3-C1059S
Σ
AT->GC
CG->TA
AT->CG
CG->AT
CG->GC
AT->TA
B
0.0
0.2
0.4
0.6
0.8
1.0
WT
met18-410C
met18Δ
pol3-C1059S
Σ
AT->GC
CG->TA
AT->CG
CG->AT
CG->GC
AT->TA
C
0.0
0.2
0.4
0.6
0.8
1.0
WT
met18Δ
AT->GC
CG->TA
AT->CG
CG->AT
CG->GC
AT->TA

Table S4. Location of mutations in 5' region (1-804) and 3' regions (805-1636 or 805-1773) of *CAN1-WT* and *CAN1-GC*[1].

Genotype	Assayed gene	Temp. (^{0} C.)	Interval in *CAN1*	Number of single bp changes	Number of deletions + insertions ≥5 bp	Number of one bp deletions + insertions	Number of complex mutations	Total number of mutations in interval
WT	*CAN1-WT*	30	1-804	20	0	6	1	27
WT	*CAN1-WT*	30	805-1636	20	2	4	3	29
WT	*CAN1-WT*	30	805-1773	20	2	4	3	29
met18-Δ	*CAN1-WT*	30	1-804	6	16	1	3	26
met18-Δ	*CAN1-WT*	30	805-1636	2	23	0	1	26
met18-Δ	*CAN1-WT*	30	805-1773	2	24	0	1	27
met18-410C	*CAN1-WT*	30	1-804	2	3	4	0	9
met18-410C	*CAN1-WT*	30	805-1636	6	2	2	1	11
met18-410C	*CAN1-WT*	30	805-1773	6	2	2	1	11
WT	*CAN1-GC*	30	1-804	23	5	13	1	42
WT	*CAN1-GC*	30	805-1636	39	1	10	6	56
WT	*CAN1-GC*	30	805-1773	39	1	10	6	56
met18-Δ	*CAN1-GC*	30	1-804	0	50	1	0	51
met18-Δ	*CAN1-GC*	30	805-1636	0	4	0	0	4
met18-Δ	*CAN1-GC*	30	805-1773	0	5	0	0	5

[1]This table shows the distribution of various types of mutations in the *CAN1* gene with wild-type base composition (*CAN1-WT*) and in a *CAN1* (*CAN1-GC*) gene in which the 5' half (bases 1-804) was GC-rich and the 3' half (bases 805-1773) had the wild-type GC content. Above, we show the number of mutations in two 3' regions 805-1773 (the region between the GC-rich region of the gene and the end of the gene) and 805-1636 (the region between the GC-rich portion of the gene and the position of most terminal nonsense codon); the rationale for the selection of these two regions is described in the text. Deletions that span the breakpoints of the various regions were not counted in this table.

Table S5. Numbers of different classes of mutations in wild-type and mutant strains.

Genotype	Assayed gene[1]	Temp. (^{0} C.)	Total number of mutations	Number of single base mutations	Number of deletions (insertions) ≥5bp[2]	Number of deletions (insertions) <5bp[2]	Number of *ura3-GC-366TC* mutations[3]	Number of other mutations
WT	*URA3-GC*	30	91	47	23 (6)	10 (0)	0	5
met18-410C	*URA3-GC*	30	44	0	38 (0)	0 (0)	6	0
met18Δ	*URA3-GC*	30	75	0	61 (0)	0 (0)	12	2
met18Δ mlh1Δ	*URA3-GC*	30	39	15	12 (0)	5 (0)	7	0
WT	*URA3-WT*	30	75	61	0 (0)	6 (1)	NR	7
met18-410C	*URA3-WT*	30	79	19	51 (1)	3 (1)	NR	4
met18Δ	*URA3-WT*	30	63	18	36 (3)	2 (0)	NR	4
met18Δ rev3Δ	*URA3-WT*	30	109	23	80 (0)	1 (0)	NR	5
WT	*CAN1-WT*	30	56	40	2 (1)	8 (2)	NR	3
met18-410C	*CAN1-WT*	30	20	8	5 (0)	3 (3)	NR	1
met18Δ	*CAN1-WT*	30	54	8	41 (0)	1 (0)	NR	4
WT	*CAN1-GC*	30	101	64	7 (0)	22 (1)	NR	7
met18Δ	*CAN1-GC*	30	61	0	60 (0)	1 (0)	NR	0
pol3-C1059S	*URA3-GC*	30	74	1	68 (0)	0 (0)	5	0
pol3-C1059S	*URA3-WT*	30	94	38	47 (1)	2 (2)	NR	4

pol3-C1059S	*CAN1-WT*	30	53	23	23 (1)	5 (0)	NR	1
pol3-13	*URA3-GC*	23	74	1	51 (0)	1 (0)	18	3
pol3-C1069S	*URA3-GC*	23	109	8	82 (1)	1 (1)	14	2
pol3-C1059S	*URA3-GC*	23	46	2	38 (0)	0 (0)	6	0
met18Δ	*URA3-GC*	23	59	1	50 (0)	0(0)	7	1
pol2-C677S	*URA3-GC*	30	55	23	15 (5)	12 (0)	0	0
pol2-C677S	*URA3-GC*	23	97	31	47 (8)	6 (1)	0	4
pol2-C2181S	*URA3-GC*	30	64	24	28 (3)	9 (0)	0	0
pol2-C763S	*URA3-GC*	23	105	57	15 (2)	20 (2)	2	7
pol2-C665S/C677S	*URA3-GC*	23	55	27	11 (2)	8 (1)	0	6
pol32Δ	*URA3-GC*	23	85	2	73 (0)	0 (0)	9	1
pol32Δ	*URA3-GC*	30	65	7	44 (1)	0 (0)	13	0

[1]The different *URA3* alleles and the strain genotypes are described in the text.

[2]Deletions and insertions are shown outside and inside parentheses, respectively.

[3]In those boxes labeled NR (not relevant), the reporter gene did not contain a quasi-palindrome that could be used as a template to generate the *ura3-GC-366TC* mutation.
